# Activation of PI3K, Akt, and ERK during early rotavirus infection leads to V-ATPase-dependent endosomal acidification required for uncoating

**DOI:** 10.1371/journal.ppat.1006820

**Published:** 2018-01-19

**Authors:** Mahmoud Soliman, Ja-Young Seo, Deok-Song Kim, Ji-Yun Kim, Jun-Gyu Park, Mia Madel Alfajaro, Yeong-Bin Baek, Eun-Hyo Cho, Joseph Kwon, Jong-Soon Choi, Mun-Il Kang, Sang-Ik Park, Kyoung-Oh Cho

**Affiliations:** 1 Laboratory of Veterinary Pathology, College of Veterinary Medicine, Chonnam National University, Gwangju, Republic of Korea; 2 Division of Life Science, Korea Basic Science Institute, Gwahak-ro, Yuseong-gu, Daejeon, Republic of Korea; Instituto de Biotecnologia, MEXICO

## Abstract

The cellular PI3K/Akt and/or MEK/ERK signaling pathways mediate the entry process or endosomal acidification during infection of many viruses. However, their roles in the early infection events of group A rotaviruses (RVAs) have remained elusive. Here, we show that late-penetration (L-P) human DS*-*1 and bovine NCDV RVA strains stimulate these signaling pathways very early in the infection. Inhibition of both signaling pathways significantly reduced production of viral progeny due to blockage of virus particles in the late endosome, indicating that neither of the two signaling pathways is involved in virus trafficking. However, immunoprecipitation assays using antibodies specific for pPI3K, pAkt, pERK and the subunit E of the V-ATPase co-immunoprecipitated the V-ATPase in complex with pPI3K, pAkt, and pERK. Moreover, Duolink proximity ligation assay revealed direct association of the subunit E of the V-ATPase with the molecules pPI3K, pAkt, and pERK, indicating that both signaling pathways are involved in V-ATPase-dependent endosomal acidification. Acidic replenishment of the medium restored uncoating of the RVA strains in cells pretreated with inhibitors specific for both signaling pathways, confirming the above results. Isolated components of the outer capsid proteins, expressed as VP4-VP8* and VP4-VP5* domains, and VP7, activated the PI3K/Akt and MEK/ERK pathways. Furthermore, psoralen-UV-inactivated RVA and CsCl-purified RVA triple-layered particles triggered activation of the PI3K/Akt and MEK/ERK pathways, confirming the above results. Our data demonstrate that multistep binding of outer capsid proteins of L-P RVA strains with cell surface receptors phosphorylates PI3K, Akt, and ERK, which in turn directly interact with the subunit E of the V-ATPase to acidify the late endosome for uncoating of RVAs. This study provides a better understanding of the RVA*-*host interaction during viral uncoating, which is of importance for the development of strategies aiming at controlling or preventing RVA infections.

## Author summary

Viral particles must transport their genome into the cytoplasm or the nucleus of host cells to initiate successful infection. Knowledge of how viruses may pirate host cell signaling cascades or molecules to promote their own replication can facilitate the development of antiviral drugs. Group A rotavirus (RVA) is a major etiological agent of acute gastroenteritis in young children and the young of various mammals. RVA enters cells by a complex multistep process. However, the cellular signaling cascades or molecules that facilitate these processes are incompletely understood. Here, we demonstrate that infection with late-penetration RVA strains results in phosphorylation of PI3K, Akt, and ERK signaling molecules at an early stage of infection, a process mediated by the multistep binding of RVAs outer capsid proteins. Specific inhibitors for PI3K/Akt and MEK/ERK signaling pathways trap the viral particles in late endosome, and acidic replenishment restores and releases them. Moreover, the RVA-induced phosphorylated PI3K, Akt, and ERK directly interact with the subunit E of the V-ATPase proton pump, required for endosomal acidification and RVA uncoating. Understanding how RVA-induced early activation of cellular signaling molecules mediates the V-ATPase-dependent endosomal acidification required for uncoating of viral particles opens up opportunities for targeted interventions against rotavirus entry.

## Introduction

Group A rotavirus (RVA), a species of the *Rotavirus* genus in the *Reoviridae* family, is recognized as a major pathogen that causes severe acute dehydrating diarrhea in young children and in a wide variety of young animals [1, 2]. RVA infections are responsible for approximately 200,000 deaths every year in children under the age of 5 years [3]. RVA is composed of a triple*-*layered particle (TLP), which surrounds eleven genomic segments of double*-*stranded RNA (dsRNA) [1, 2]. RVAs enter the cell by a complex multistep process in which different domains of the RVA surface proteins, including the VP8* and VP5* domains of the spike VP4 protein, and the VP7 protein, interact with different cell surface receptors [4, 5]. Several lines of evidence indicate that most RVAs enter the cell by clathrin-mediated endocytosis [4, 6, 7], although some RVAs, including rhesus rotavirus (RRV), are known to enter the cell through a clathrin- and caveolin-independent pathway [4, 8, 9].

Following virus uptake, RVAs travel to different endosomal compartments before uncoating and release of a double-layered particle (DLP) into the cytosolic space. Uncoating and release of DLP are triggered either in the maturing endosome (ME) [early-penetrating (E-P) viruses] or in the late endosome (LE) [late-penetrating (L-P) viruses] depending on the process requirements, which differ between RVA strains [9–12]. For RVA uncoating to deliver DLP into cytoplasm, the environment of the RVA-containing endosomes has to change, for instance, by endosomal acidification, a drop in calcium concentration, the exchange of membrane components, the formation of additional intraluminal vesicles, or the acquisition of lysosomal components [4, 13]. Among these mechanisms, endosomal acidification plays a crucial role in RVA uncoating of L-P strains such as the human strain Wa, the porcine strain TFR-1 and the bovine strain UK, but not in uncoating of E-P strains such as RRV [4, 11, 14].

The vacuolar-H^+^ ATPase (V-ATPase) proton pump is a multi-subunit membrane protein complex, which is found in the membranes of intracellular organelles and in the plasma membrane of certain specialized cells. It is composed of a peripheral V_1_ domain (consisting of subunits A to H), mediating the hydrolysis of ATP, and a membrane-bound V_0_ domain (consisting of subunits a, c, c', c", d, and e), translocating protons across the membrane. It maintains the acidification of endosomes, lysosomes, phagosomes and Golgi-derived secretory vesicles [15]. Recently, the V-ATPase has been found to be involved in cell entry of viruses by maintaining the acidic pH within the endosome necessary for viral genome release [16]. The acidic milieu of LE constitutes a critical precondition that induces uncoating of a wide variety of enveloped and non-enveloped viruses including L-P strains [4, 17].

The phosphoinositide 3-kinase/protein kinase B [known as Akt] (PI3K/Akt) signaling pathway regulates a wide range of cellular processes such as mitogenic signaling, cell survival, proliferation, apoptosis, and cytoskeleton remodeling [18, 19]. The mitogen-activated protein-extracellular signal-regulated kinase/extracellular-regulated kinase (MEK/ERK) signaling cascade mediates a large variety of processes including cell adhesion, cell cycle progression, cell migration, cell survival, differentiation, metabolism, proliferation, and transcription [20]. Many viruses, including DNA and RNA viruses, employ the PI3K/Akt and/or MEK/ERK pathways to facilitate different stages of their life cycle [18–21]. With regard to the initial stages of viral infection, the signaling pathways or their molecules participate in virus entry and/or uncoating. For example, these signaling pathways mediate the viral entry process of many viruses such as hepatitis C virus (HCV) [22], Ebola virus [23], and human rhinovirus serotype 2 [24], whereas influenza A virus (IAV) employs both PI3K and ERK to mediate V-ATPase-dependent endosomal acidification for fusion [25].

Activation of the PI3K signaling pathway has been found to play a crucial role in the increased yield of infectious RVAs by improving the adhesion and survival of infected cells [26]. The heat shock protein-90 (Hsp90), a molecular chaperone, is also known to modulate RVA replication during the late stage of the virus life cycle through activation of the PI3K/Akt signaling pathway [27]. The RVA-induced cellular apoptosis observed during the early stage of infection is inhibited by the RVA non-structural protein 1 via activation of the PI3K/Akt and NF-κB prosurvival pathways [28–30]. In addition, RVA activates the COX-2/PGE_2_ axis by modulating the MEK/ERK pathway which promotes RVA replication [31].

While the PI3K/Akt and/or MEK/ERK signaling cascades have been found to play different roles in the entry and/or uncoating of various viruses, their role in RVA entry and uncoating has remained elusive. Here, we demonstrate that the immediate early activation of the molecules PI3K, Akt, and ERK is important for endosomal acidification and uncoating of the L-P strains DS-1 and NCDV. Inhibition of both cascades blocked the release of both DS*-*1 and NCDV strains from the LE with a reduction in the fluorescence intensity of the CMFDA pH probe. Moreover, the direct interaction of phosphorylated PI3K, Akt, and ERK molecules with the subunit E of the V-ATPase V_1_ domain in response to early RVA infection emphasize the importance of RVA-induced PI3K, Akt, and ERK early activation as signaling mediators of the V-ATPase-stimulated endosomal acidification required for RVA uncoating.

## Results

### Activation of PI3K/Akt and MEK/ERK signaling pathways during immediate early RVA infection

**Fig 1:**
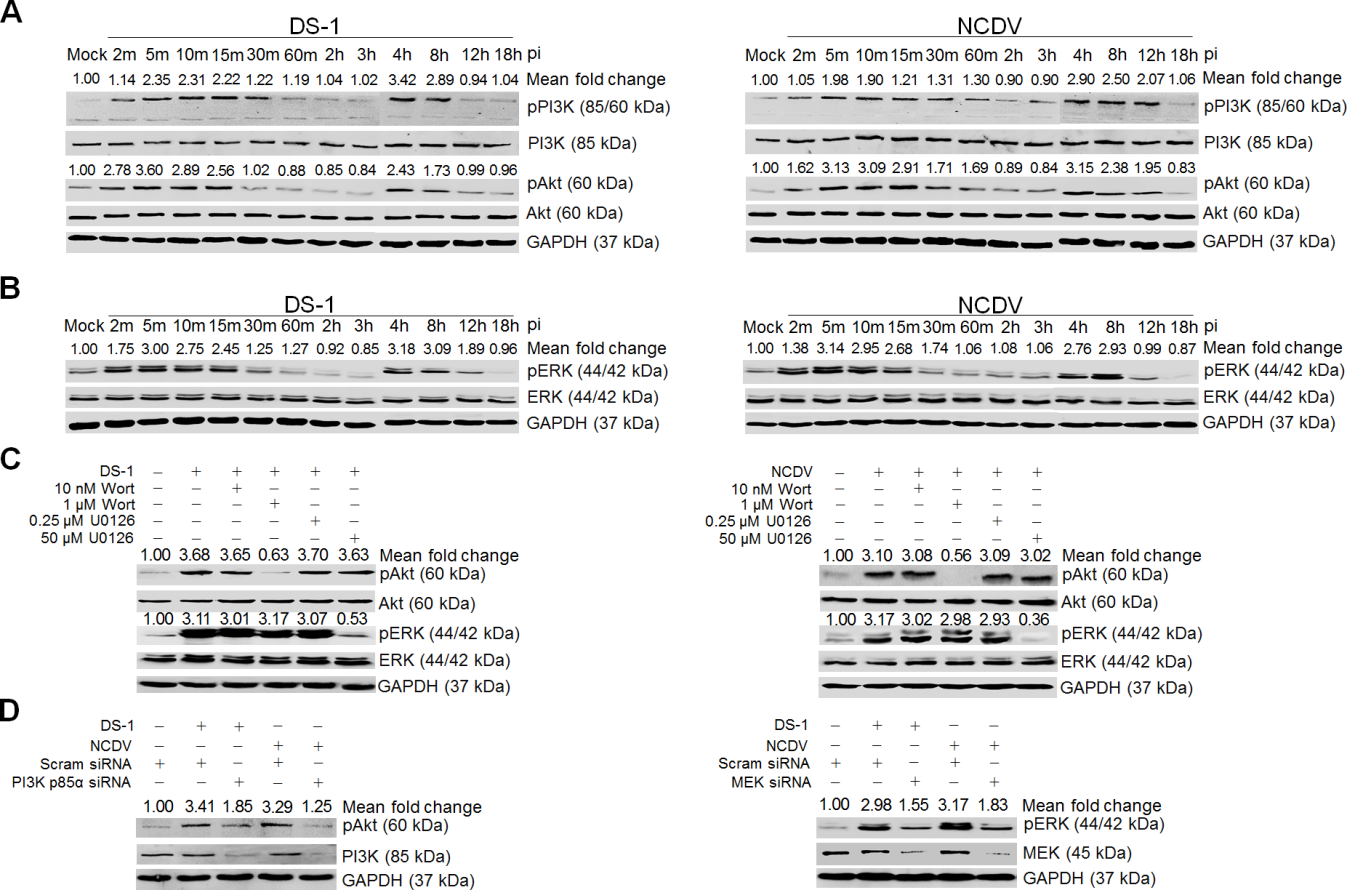
RVA-induced early activation of PI3K, Akt, and ERK signaling molecules in MA104 cells. The human RVA DS-1 (A) and bovine RVA NCDV (B) strains (MOI = 10 FFU/cell) were inoculated into serum*-*starved MA104 cells. Cells were harvested at the indicated time points. Cell lysates were subjected to Western blot analysis to check the expression levels of phosphorylated PI3K (pPI3K), PI3K, pAkt, Akt, pERK, and ERK using the relevant antibody. GAPDH was used as a loading control. (C) MA104 cells were mock*-*treated or pretreated with wortmannin or U0126 at the indicated doses for 1 h at 37°C, followed by infection with strains DS-1 or NCDV. Cell lysates were harvested at 5 mpi and the expression levels of pAkt, Akt, pERK, and ERK were evaluated by Western blot analysis. GAPDH was used as a loading control. (D) MA104 cells were transfected with scrambled siRNA or siRNAs specific for PI3K p85α or MEK and then infected with the DS-1 and NCDV strains (MOI = 10 FFU/cell). Cell lysates were subjected to Western blot analysis to check the expression levels of pAkt, Akt, pERK, and ERK using the appropriate antibody. GAPDH was used as a loading control. The intensity of pPI3K, pAkt, and pERK relative to GAPDH was determined by densitometric analysis and is indicated above each lane.

To explore whether the PI3K/Akt and MEK/ERK signaling pathways are activated during immediate early RVA infection, monkey kidney epithelial MA104 cells and human intestinal epithelial Caco-2 cells were mock infected or infected with the human RVA strain DS-1 or the bovine RVA strain NCDV at a MOI of 10 for the indicated time points. Each mock*-* or RVA*-*infected cell lysate was subjected to Western blot analysis with antibodies specific for PI3K, phosphorylated PI3K (pPI3K), Akt, phosphorylated Akt (pAkt), ERK, and phosphorylated ERK (pERK). In comparison with mock-inoculated cells, increased phosphorylation of the signaling molecules PI3K, Akt, and ERK was detected in RVA-infected cells as early as 2 min after virus inoculation, with a maximum at 5 min that was sustained at high level until 15 min, and declined afterwards (Fig 1 and S1 Fig). In addition, both signaling pathways were reactivated at 4 h post-infection (hpi) and remained active up to 12 hpi (Fig 1 and S1 Fig). We next determined whether the simian RVA strain RRV, which is considered an E-P strain [10, 12], similarly upregulates pPI3K, pAkt, and pERK during entry in MA104 and Caco-2 cells. Interestingly, the RRV strain activated both signaling pathways at 30 and 60 min post-infection (mpi) in MA104 and Caco-2 cells (S2 Fig), respectively, with activation patterns similar to those previously reported [26].

We next checked whether phosphorylation of Akt and ERK is mediated by the upstream signaling molecules PI3K and MEK, and whether the PI3K/Akt and MEK/ERK pathways influence each other during immediate early RVA infection. MA104 and Caco-2 cells were pretreated with inhibitors specific for PI3K (wortmannin) and MEK (U0126). Each inhibitor specifically and efficiently reduced the levels of the corresponding downstream molecule in virus-infected cells in comparison with the levels in mock-treated and virus-inoculated cells (Fig 1C and S1C Fig). Furthermore, knockdown of PI3K p85α and MEK by transfection with specific siRNAs resulted in a reduction in pAkt and pERK, respectively, following RVA infection (Fig 1D and S1D Fig). Our results suggest that, in contrast to the simian RVA E-P strain RRV [10, 12], both the human and animal RVA strains DS-1 and NCDV concomitantly and independently activate the signaling pathways PI3K/Akt and MEK/ERK during immediate early RVA infection. Since MA104 cells are reasonably transfectable, highly permissive for many RVA strains, and have been widely used in the study of RVA entry [12], we used this cell line in the rest of the experiments.

### Involvement of PI3K/Akt and MEK/ERK signaling pathways in RVA entry

Activation of the PI3K/Akt and MEK/ERK pathways during immediate early RVA infection might be involved in the RVA life cycle and blocking these pathways might eventually influence virus replication [26–31]. To further investigate whether these signaling pathways are involved in the RVA life cycle, we pretreated cells for 1 h with wortmannin, followed by RVA inoculation and incubation for 8 h, and we then checked the infectivity of the viral progeny. Compared to the mock control, pretreatment with wortmannin reduced total viral RNA in a dose-dependent manner (S3A Fig), resulting in a dose-dependent gradual decline in virus-infected cells (S3B and S3C Fig), viral protein expression (S3D Fig) and infectivity of viral progeny (S3E Fig). Likewise, pretreatment of MA104 cells with U0126 reduced the infectivity of the progeny of both human and animal RVA strains (S4 Fig). The reduction of the RVA life cycle was not due to cytotoxicity of the inhibitors used as no apparent effect of the chemicals was found on MA104 cell viability (S5 Fig). Taken together, these data suggest that immediate early induction of both PI3K/Akt and MEK/ERK signaling pathways could play a role in one or more steps of the RVA replication cycle.

### Role of late endosomal acidification in rotavirus entry

**Fig 2:**
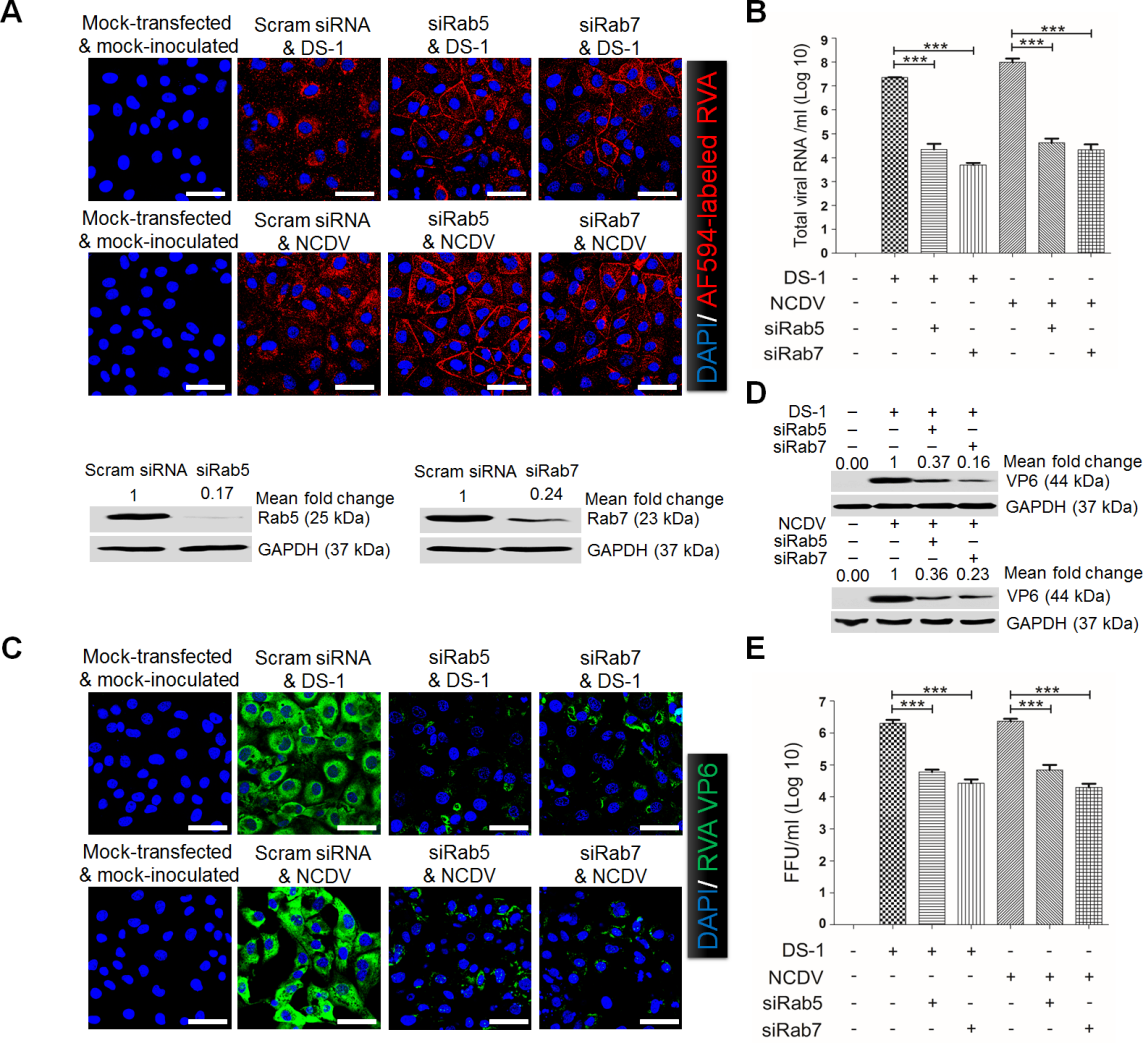
Rotavirus entry and infection depend on Rab5 and Rab7. (A) MA104 cells were transfected with either scrambled siRNA or siRNAs against Rab5 or Rab7. Afterwards, cells were exposed to Alexa 594-labeled DS*-*1 (approximately 595 particles/cell) or NCDV (approximately 790 particles/cell) for 30 min at 4°C. Unbound virus was washed off and the cells were shifted to 37°C for 90 min. Cells were then fixed and processed for confocal microscopy. Cells treated in parallel were analyzed by Western blot analysis to ensure effective knockdown of protein levels. (B*-*E) MA104 cells transfected with siRNAs against Rab5 or Rab7 were infected with the DS-1 and NCDV strains (MOI = 10 FFU/cell). The total viral RNA (B), antigen*-*positive cells (using anti*-*RVA VP6 Mab) (C), and VP6 protein (D) were determined by real*-*time RT*-*PCR, immunofluorescence, and Western blot analyses, respectively. GAPDH was used as a loading control. The intensity of pPI3K, pAkt, and pERK relative to GAPDH was determined by densitometric analysis and is indicated above each lane. (E) The virus titer was determined by cell culture immunofluorescence assay using cell lysates produced by 3 cycles of freezing and thawing, and is expressed as FFU. All experiments were performed in triplicate and panels A and C show a representative set of results. Data are presented as means ± standard error of the mean from three independent experiments. Differences were evaluated using the One-Way ANOVA. *p<0.05; **p<0.001; ***p<0.0001. The scale bars in panels A and C correspond to 20 μm.

The above results indicated that the activation of the PI3K/Akt and MEK/ERK pathways detected during the immediate early phase of RVA infection might be involved in RVA entry. Before examining the involvement of these pathways in RVA entry, we first analyzed endosomal trafficking of the human DS-1 strain and the bovine NCDV strain; the former is already known to use both early endosomes (EEs) and LEs [10]. In MA104 cells transfected with scrambled siRNA, both the human and the bovine RVA strains labeled with Alexa Fluor 594 (AF594) were mainly localized in the perinuclear area (Fig 2A). However, silencing of an EE marker Ras-related protein 5 (Rab5), or a LE marker Rab7 by transfection with specific siRNAs trapped the AF594-labeled viruses in the periphery of the cytoplasm (Fig 2A). Blocking of intracellular trafficking by these siRNAs resulted in a reduction of total viral RNA, virus*-*infected cell numbers, viral protein expression, and infectivity of the viral progeny in comparison with mock*-*treated, virus*-*infected cells (Fig 2B*–*2E). Our results are consistent with previous studies showing that L-P RVAs, such as the human DS-1 and the bovine NCDV strains used in this study, enter the cell through both EEs and LEs [4, 10].

**Fig 3:**
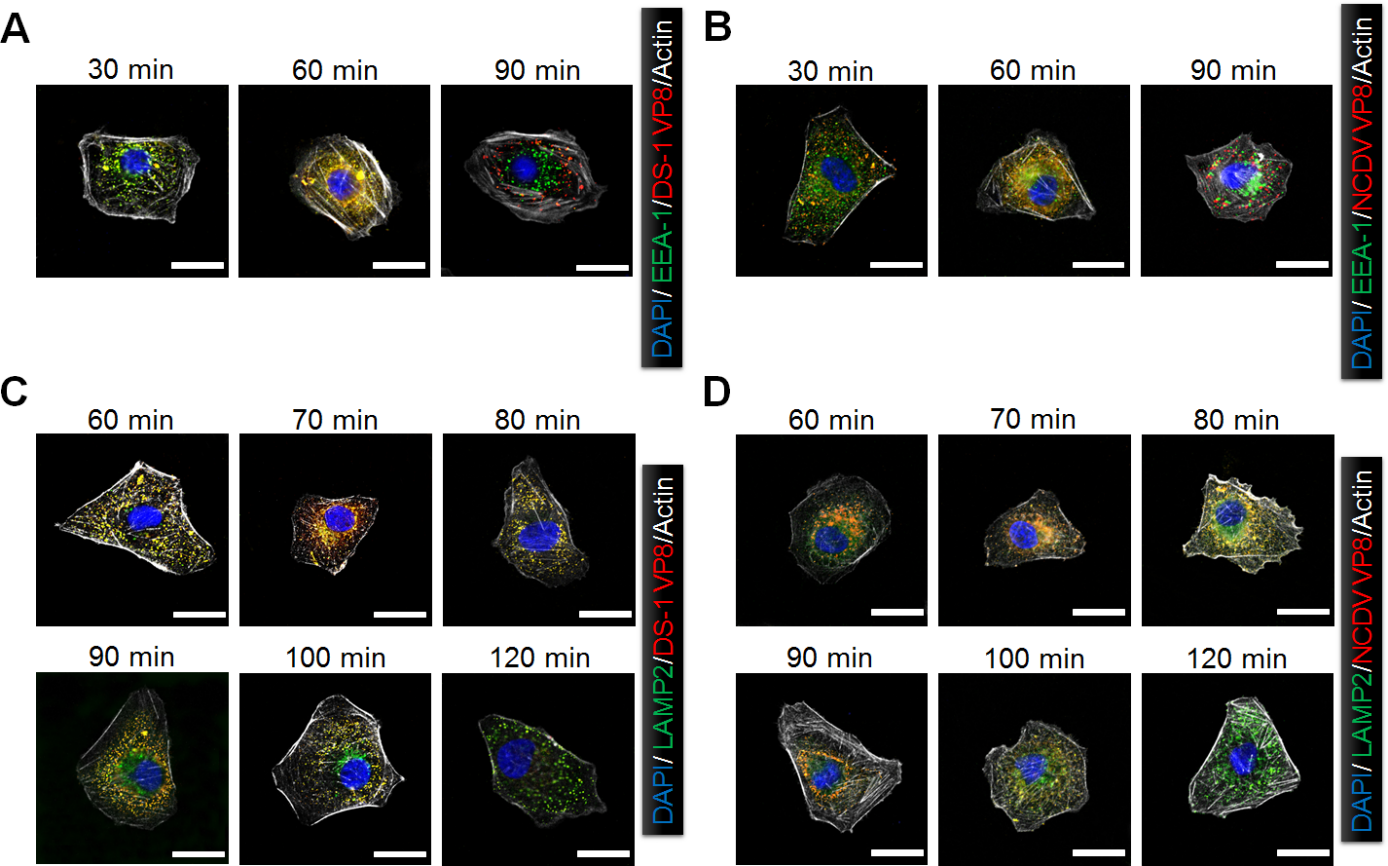
Rotavirus transits from the early to the late endosome during the internalization process. MA104 cells were incubated with the DS-1 (A and C) and NCDV (B and D) strains for the indicated times at 37°C. The cells were then fixed, permeabilized, and processed for immunofluorescence assay. Virus particles were visualized using anti-VP8* primary antibody and secondary antibody labeled with Alexa Fluor 594. Endosomal markers were detected with specific antibodies against EEA1 (A and B) and LAMP2 (C and D), and the corresponding Alexa Fluor 647-conjugated secondary antibodies. Actin cytoskeleton was stained with Alexa Fluor 488-labeled phalloidin. All experiments were performed in triplicate and panels A to D show a representative set of results. The scale bars in each panel correspond to 5 μm.

To further confirm the above results and to study the dynamics of entry of both strains, we examined the colocalization of viral particles (using anti*-*VP8* antibody) with the EE marker early endosome antigen 1 *(*EEA1) and the LE marker lysosome*-*associated membrane protein-2 (LAMP2) at different time points. Colocalization of DS*-*1 and NCDV viral particles with EEA1 gradually increased, reaching a maximum at 60 mpi and decreasing thereafter (Fig 3A and 3B). Colocalization of both viral particles with LAMP2 was found to increase gradually starting at 60 mpi (Fig 3C and 3D). Interestingly, the time required to attain the maximum signal was found to be different for each strain. The maximum colocalization signal of NCDV particles with LAMP2 was achieved at 80 mpi, and it started to decline at 90 mpi (Fig 3D). However, DS*-*1 had a maximum colocalization with LAMP2 at 90 mpi (Fig 3C), suggesting that the uncoating time was between 80 and 90 mpi for NCDV and between 90 and 100 mpi for DS*-*1. The loss of VP8* staining at 120 mpi (Fig 3) suggested that decapsidation of the virions, i.e. uncoating, had already occurred.

**Fig 4:**
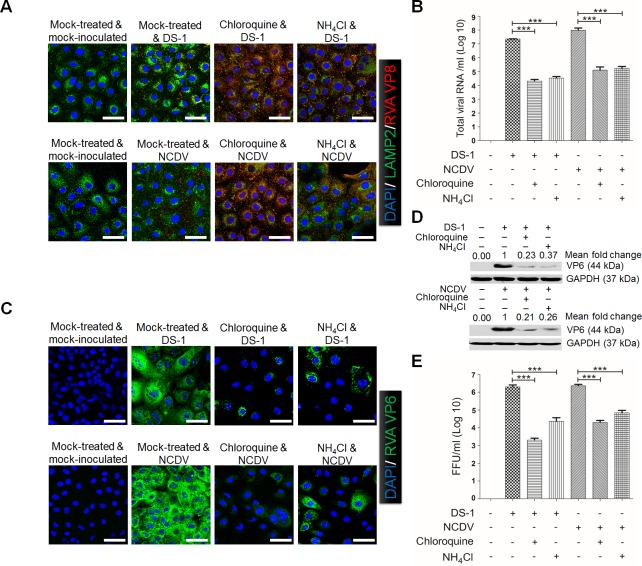
Rotavirus requires endosomal acidification for efficient internalization. (A) Mock-, 100 μM chloroquine-, or 100 mM ammonium chloride (NH_4_Cl)-pretreated MA104 cells were incubated with the DS*-*1 or NCDV strains for 30 min at 4°C and then shifted to 37°C for 2 h. The cells were then fixed and processed for immunofluorescence using anti*-*VP8* primary antibody in parallel with anti*-*LAMP2 antibody. (B-D) RVA DS*-*1 and NCDV strains (MOI = 10 FFU/cell) were inoculated into mock- or chloroquine- or NH_4_Cl-pretreated MA104 cells. The total viral RNA (B), antigen*-*positive cells (using anti*-*RVA VP6 Mab) (C), and VP6 protein (D) were determined by real*-*time RT*-*PCR, immunofluorescence, and Western blot analyses, respectively. GAPDH was used as a loading control. The intensity of pPI3K, pAkt, and pERK relative to GAPDH was determined by densitometric analysis and is indicated above each lane. (E) The virus titer was determined by immunofluorescence assay using cell lysates produced after 3 cycles of freezing and thawing, and it is expressed as FFU. All experiments were performed in triplicate and panels A and C show a representative set of results. Data are presented as means ± standard error of the mean from three independent experiments. Differences were evaluated using the One*-*Way ANOVA. *p<0.05; **p<0.001; ***p<0.0001. The scale bars in panels A and C correspond to 20 μm.

The above results showed that both strains reached the LEs and that the uncoating process was completed within 120 mpi. Therefore, we next assessed whether the uncoating process of both strains are mediated by acidification of LEs. For this purpose, we employed chloroquine and ammonium chloride (NH_4_Cl) which inhibit endosomal acidification. As expected, the spots of VP8* antigens colocalized with LAMP2 had disappeared in the cytoplasm at 120 mpi (Fig 4A). However, both chemical inhibitors retained both strains in LAMP2-positive LEs in the cytoplasm (Fig 4A). As a consequence of virus trapping in LEs, total viral RNA, virus*-*infected cell numbers, viral protein expression, and infectivity of viral progeny were significantly reduced in comparison with mock*-*treated, virus*-*infected cells (Fig 4B–4E). These data suggested that both strains were uncoated in the acidified LE.

### Inhibition of PI3K/Akt and MEK/ERK pathways blocks the release of L-P RVA strains from late endosome by affecting endosomal acidification

**Fig 5:**
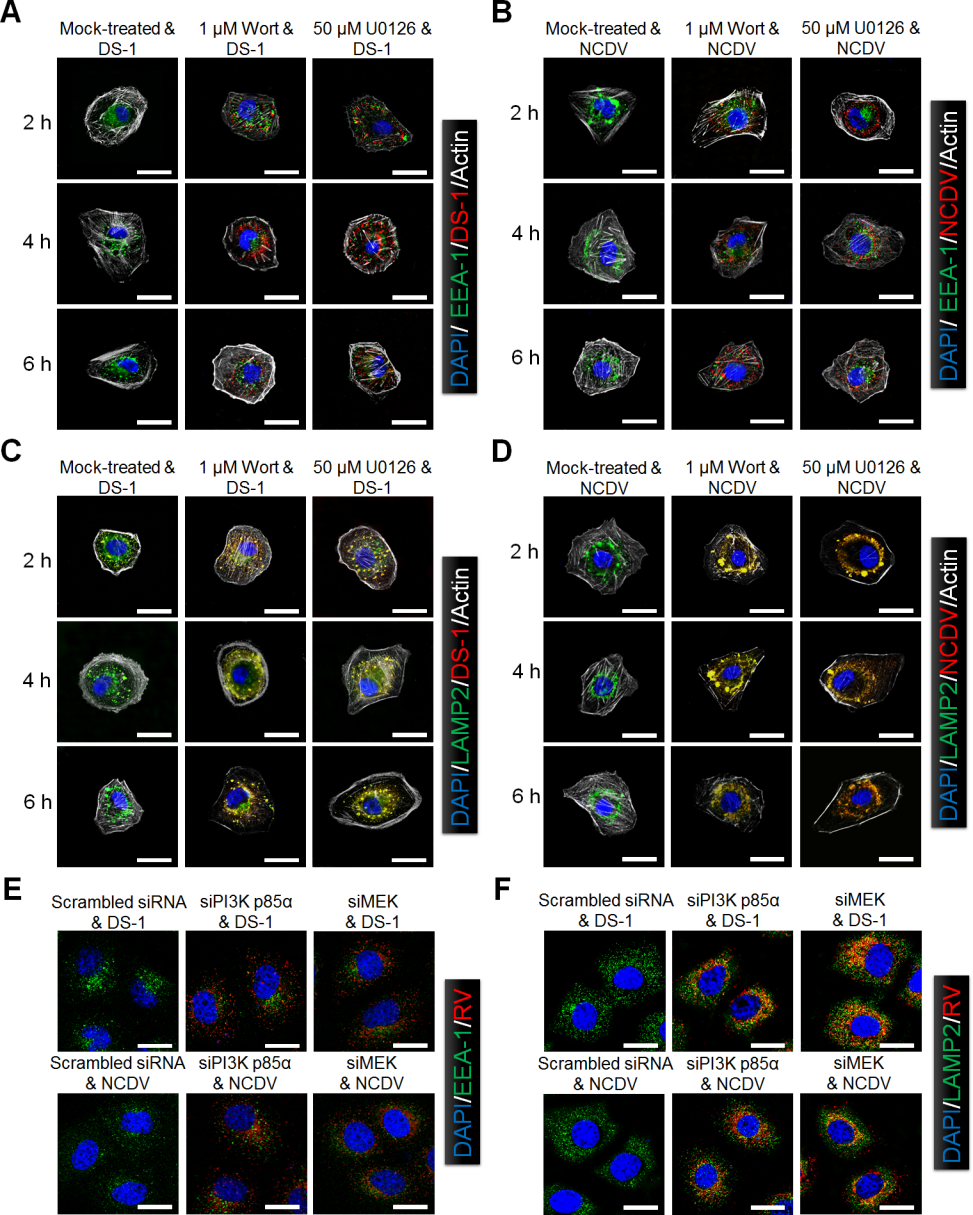
Involvement of PI3K/Akt and MEK/ERK signaling pathways in RVA uncoating. (A-D) MA104 cells were pretreated with or without wortmannin or U0126 for 1 h at 37°C and then infected with the DS-1 or NCDV strains (MOI = 10 FFU/cell) for the indicated time. After fixation and permeabilization, the cells were prepared for confocal microscopy using anti*-*VP8* antibody, anti*-*EEA1 antibody (A and B), and anti-LAMP2 antibody (C and D), and the relevant secondary antibodies. Actin cytoskeleton was stained with AF488-labeled phalloidin. (E and F) MA104 cells were transfected with scrambled siRNA or siRNAs specific for PI3K p85α or MEK, and then infected with the DS-1 and NCDV strains (MOI = 10 FFU/cell). After fixation and permeabilization, the cells were prepared for confocal microscopy using anti*-*VP8* antibody, anti*-*EEA1 antibody (E), and anti-LAMP2 antibody (F), and the relevant secondary antibodies. Representative images are shown. The scale bars correspond to 5 μm.

The above data implied that PI3K/Akt and MEK/ERK signaling pathways might be involved in the RVA entry process. Next, we investigated which step(s) of the RVA entry process was influenced by these signaling pathways. To check whether both signaling pathways are involved in virus trafficking from EEs to LEs, MA104 cells were preincubated with either wortmannin or U0126, infected with DS*-*1 or NCDV for 30 min at 4°C, washed, and shifted to 37°C for the indicated time points. Subsequently, colocalization of both strains with EEA1 or LAMP2 was assessed by confocal microscopy. As expected, none of the RVA strains were detected by immune-fluorescence assay (IFA) using an antibody specific for the VP8* protein in mock-treated cells after 2 hpi since uncoating of these strains had already occurred at these time points (Fig 5). After pretreatment with either of the two chemicals, the fluorescence pattern of the DS-1 and NCDV VP8* antigens was still present but it did not colocalize with EEA1 even after 6 hpi (Fig 5A and 5B). However, neither of the chemicals affected the colocalization of viral particles with LAMP2, even when the incubation time was extended until 6 hpi (Fig 5C and 5D). We further confirmed these results by examining the effect of PI3K p85α and MEK specific siRNAs on colocalization of RV particles with EEA1 and LAMP2. As shown in Fig 5E and 5F, silencing of either PI3K p85α or MEK also trapped RVA particles in the LEs. Taken together, these data indicate that the PI3K/Akt and MEK/ERK signaling pathways do not influence trafficking of the L-P RVA strains DS-1 and NCDV from the EEs to the LEs.

**Fig 6:**
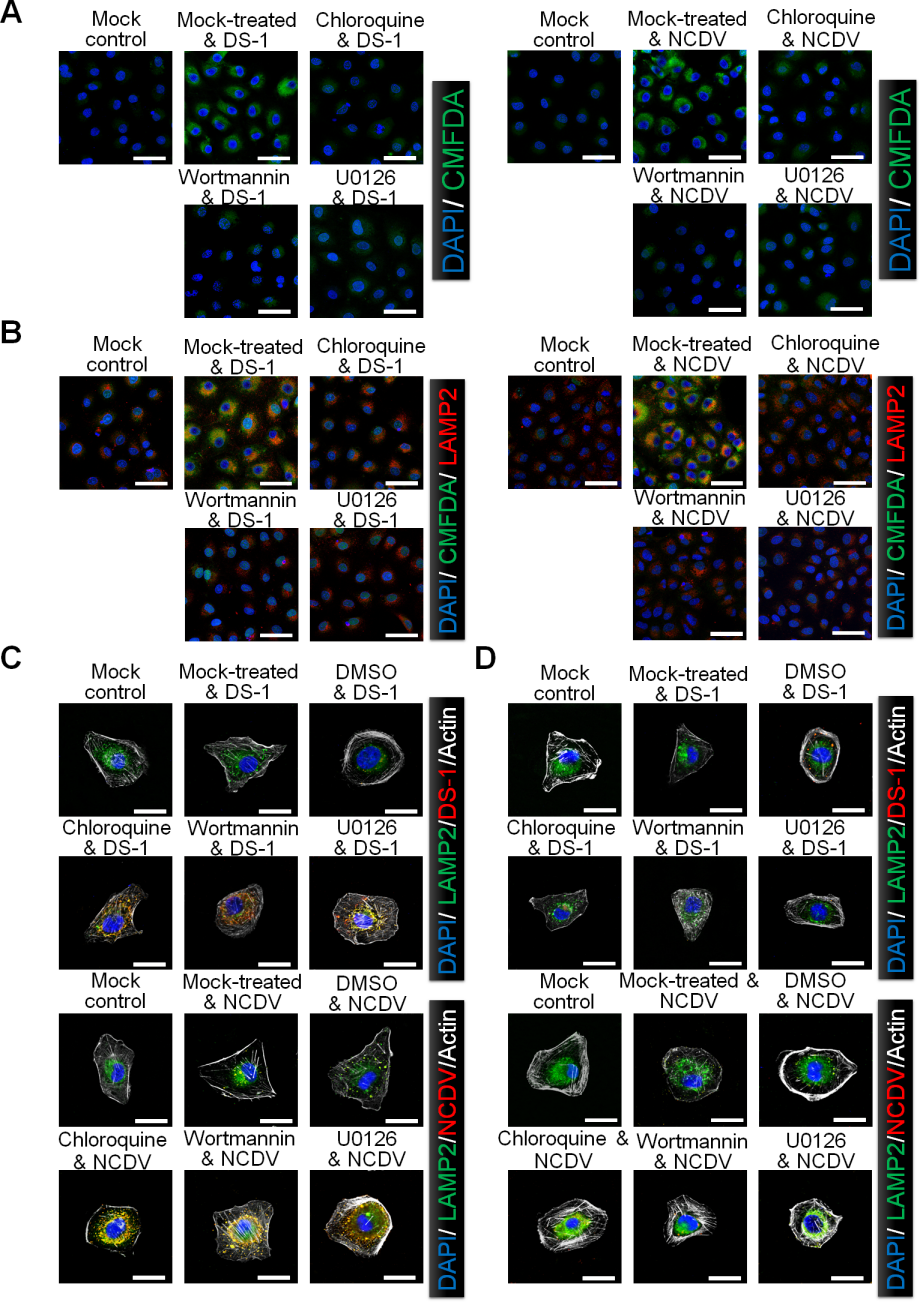
Involvement of PI3K/Akt and MEK/ERK signaling pathways in late endosomal acidification. MA104 cells were pretreated with or without chloroquine, wortmannin or U0126 for 1 h at 37°C, and subsequently infected with the strains DS*-*1 or NCDV (MOI = 10 FFU/cell) for 30 min at 37°C. Cells were then incubated for 30 min with CMFDA (10 μM) to visualize acidification of intracellular compartments followed by 30 min in serum*-*free media (A), or incubated with CMFDA followed by anti-LAMP2 antibody to check colocalization by confocal microscopy (B). (C and D) After chemical pretreatment and virus infection, cells were incubated with neutral (pH 7.2) (C) or acidic (pH 5) (D) buffers for 5 min at 37°C. The cells were then washed and incubated for further 2 h at 37°C and prepared for confocal microscopy to check colocalization of RVA VP8* with LAMP2. Representative images are shown. The scale bars correspond to 20 μm (A and B) and 5 μm (C and D).

Since both wortmannin and U0126 blocked the release of DS*-*1 and NCDV viral particles from the LE (Fig 5), both signaling pathways could be involved in acidification of the LEs for uncoating and release of DLPs. Therefore, we first examined deposition of the cell tracker CMFDA pH probe for monitoring endosomal acidification. The fluorescence intensity of the CMFDA*-*positive signal markedly increased in DS-1- and NCDV*-*infected cells compared with mock*-*infected control cells (Fig 6A). Like the inhibitory effect of chloroquine on endosomal acidification, pretreatment of MA104 cells with wortmannin or U0126 significantly eliminated the CMFDA fluorescent signal in DS-1*-* or NCDV*-*infected cells (Fig 6A). Since the E-P RVA strain RRV does not use endosomal acidification for uncoating [4, 7, 11, 14], a basal level of CMFDA fluorescence could be seen in RRV-infected MA104 cells regardless of pretreatment with wortmannin or U0126 (S6A Fig).

The CMFDA*-*positive structures were found to colocalize with LAMP2 in mock*-*treated, DS-1*-* or NCDV*-*infected cells (Fig 6B). However, the intensity of the CMFDA*-*positive signal decreased with loss of LAMP2 colocalization in virus-infected cells treated with inhibitors for endosomal acidification (chloroquine), PI3K (wortmannin), or MEK (U1026) (Fig 6B). Since trafficking of the E-P RVA strain RRV during entry into MA104 cells is restricted to the EE compartment (12), colocalization of RRV with EEA1 or LAMP2 was not observed in MA104 cells at 3 hpi after pretreatment with either PI3K (wortmannin) or MEK inhibitors (U0126) because uncoating of the RRV strain had already occurred at this time point (S6B Fig).

To confirm the above results, we next investigated whether replacement of culture medium with acidic buffer in cells treated with either of the two inhibitors restores virus release from LEs. In mock- or dimethyl sulfoxide (DMSO)*-*treated cells incubated with the strains DS*-*1 or NCDV for 2 h in medium at neutral pH, the immunofluorescence staining of viral VP8* of both NCDV and DS-1 strains disappeared, indicating that the virus was already uncoated (Fig 6C). In contrast, MA104 cells treated with chloroquine, wortmannin or U0126 showed colocalization of the viral particles with LAMP2 even after 2 h incubation (Fig 6C). Interestingly, acidic replenishment of the culture medium induced evanishment of viral particles from the LEs in chemical*-* or inhibitors*-*treated cells at 2 hpi (Fig 6D). Collectively, these findings suggest that the PI3K/Akt and MEK/ERK signaling pathways are involved in acidification of the LEs for promoting the release of RVA DLPs into the cytoplasm.

### PI3K/Akt and MEK/ERK signaling cascades mediate endosomal acidification through direct interaction with subunit E of V*-*ATPase V_1_ domain

**Fig 7:**
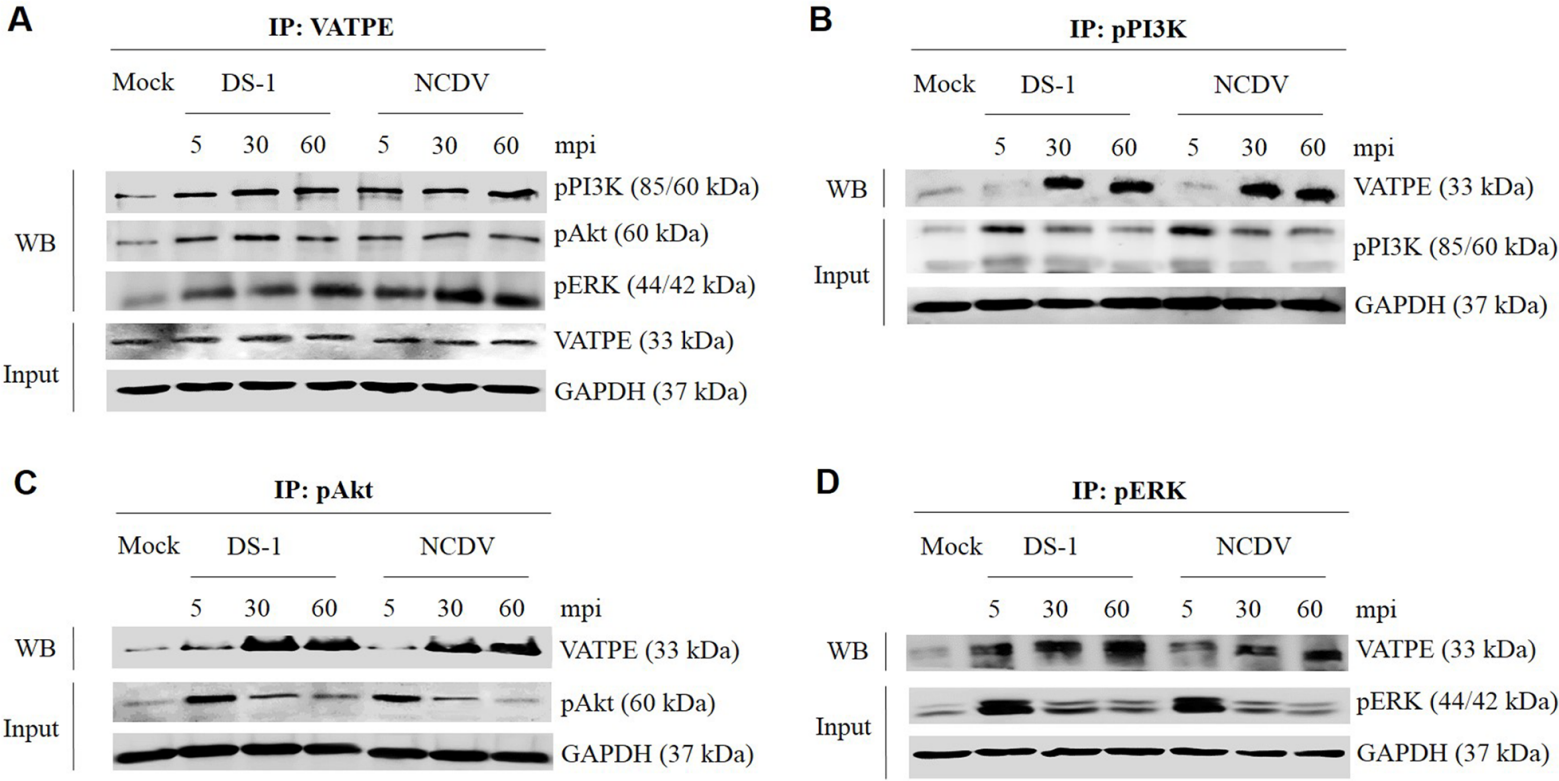
Direct interaction of pPI3K, pAkt, and pERK with subunit E of the V-ATPase V_1_ domain for endosomal acidification. (A-D) Serum-starved MA104 cells were inoculated with RVA DS-1 or NCDV (MOI = 10 FFU/cell) for the indicating time. Subsequently, the cell lysates were immunoprecipitated using antibodies specific for the V_1_ subunit E of the V*-*ATPase (A), pPI3K (B), pAkt (C), and pERK (D). The co-immunoprecipitated products were analyzed by Western blot analysis to detect pPI3K, pAkt, pERK, and the V_1_ subunit E using the relevant antibodies. GAPDH was used as a loading control.

Since the acidification mechanism of the endosomal environment could be reliant on the V-ATPase [17], we next investigated whether activation of the signaling molecules PI3K, Akt, and ERK could mediate endosomal acidification through direct interaction with the V-ATPase upon L-P RVA infection. To assess this, MA104 cells were either mock-infected or infected with the DS-1 or NCDV strains, and cell lysates were immunoprecipitated with antibodies specific for the subunit E of the V_1_ domain of the V-ATPase, pPI3K, pAkt or pERK. The antibody specific for the subunit E pulled down pPI3K, pAkt and pERK in the immunoprecipitated cell lysates (Fig 7A). Immunoprecipitation using antibodies specific for pPI3K, pAkt or pERK precipitate the subunit E of the V_1_ domain of the V-ATPase, confirming the above results (Fig 7B–7D). As a negative control, the cell lysates were immunoprecipitated with an irrelevant antibody against Na^+^/K^+^-ATPase B1 protein and then the immunoprecipitated proteins were evaluated by Western blot analysis. The results showed that antibody against Na^+^/K^+^-ATPase B1 protein did not interact with V-ATPase, pPI3K, pAkt, or pERK (S7A and S7C Fig). To further confirm the IP results, the reaction mixture containing each antibody against V-ATPase E subunit, pPI3K, pAkt, or pERK, and cell lysate was incubated with secondary antibodies specific for each primary antibodies. Afterward, the immune complexes were captured by incubation with A- or G-agarose beads and the immunoprecipitated proteins were evaluated by Western blot analysis. The results showed that all three molecules (pPI3K, pAkt, and pERK) interacted with V-ATPase (S7B and S7C Fig).

**Fig 8:**
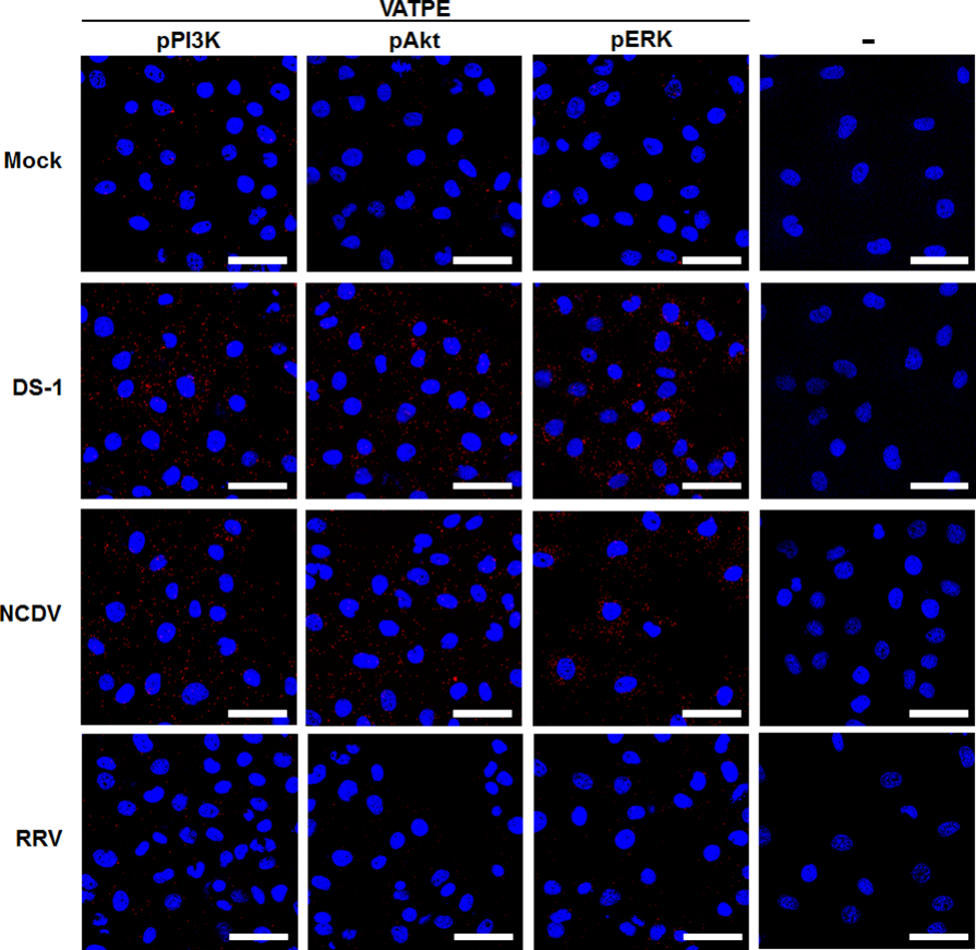
Direct interaction of pPI3K, pAkt, and pERK with the subunit E of the V-ATPase V_1_ domain determined by Duolink proximity assay. Serum-starved MA104 cells were either mock-inoculated or inoculated with the RVA strains DS-1, NCDV, and RRV (MOI = 10 FFU/cell). Subsequently, the cells were fixed, permeabilized and incubated with or without the primary antibodies (goat anti-V-ATPase E subunit and rabbit anti-pPI3K, pAkt, and pERK antibodies) overnight at 4°C. The Duolink PLA was performed as described in the Materials and Methods section and the signals are represented as red dots. Representative images are shown. The scale bars correspond to 20 μm.

To corroborate the above immunoprecipitation results, the interaction between the subunit E of the V_1_ domain of the V-ATPase and pPI3K, pAkt, and pERK was further evaluated in RVA-infected cells using Duolink proximity ligation assay (PLA). In this assay, the signal from the interaction of two proteins in close proximity (40 nm or less) is easily visible as a distinct fluorescent spot [32]. The assay was able to identify the subunit E of the V_1_ domain of the V-ATPase and pPI3K, pAkt, or pERK as partners in MA104 cells infected with either the DS-1 or the NCDV strains, as indicated by the red dots (Fig 8). In contrast, the E-P RVA strain RRV, which does not require endosomal acidification [4, 7, 11, 14], did not induce any positive signal in MA104 cells (Fig 8). As a negative control, the irrelevant antibody against Na^+^/K^+^-ATPase B1 protein did not interact with V-ATPase, pPI3K, pAkt, and pERK in the mock- or infected cells with RVA strains, DS-1, NCDV, or RRV (S8 Fig). Taken together, these results indicate that, during immediate early infection of the L-P strains DS-1 and NCDV, pPI3K, pAkt, and pERK directly interact with the subunit E of the V_1_ domain of the V-ATPase, resulting in late endosomal acidification.

### RVA outer capsid proteins can trigger the activation of PI3K/Akt and MEK/ERK pathways leading to LE acidification for uncoating of L-P RVA strains

**Fig 9:**
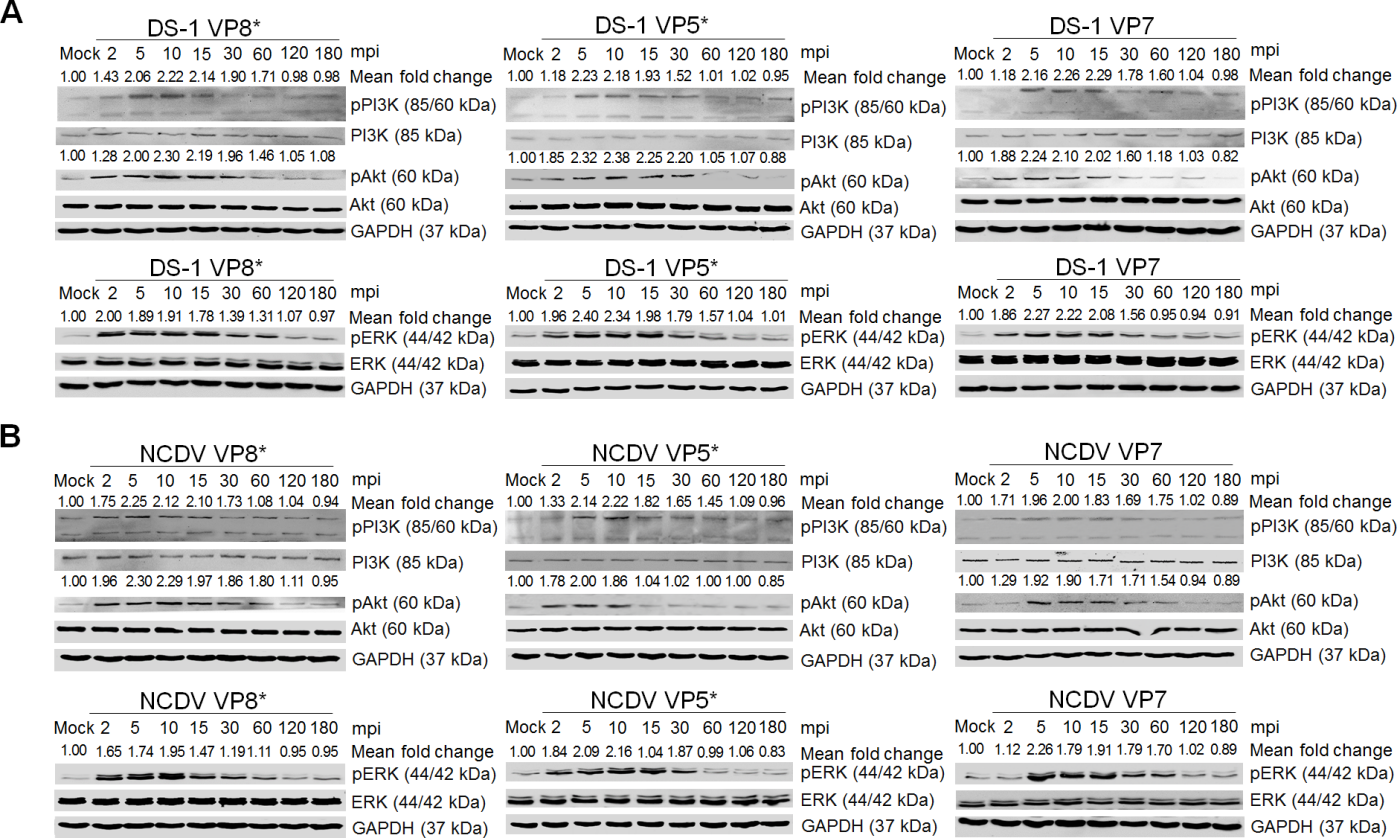
Activation of the PI3K/Akt and MEK/ERK signaling pathways by RVA outer capsid surface proteins. Serum*-*starved MA104 cells were incubated with recombinant GST-fused VP8* or his-tagged VP5* or VP7 proteins of the strains DS-1 (A) and NCDV (B) at 10 μg/ml for the indicated time points. The cell lysates were subjected to Western blot analysis for the detection of pPI3K, pAkt, pERK, PI3K, Akt, and ERK using the relevant antibodies. GAPDH was used as a loading control. The intensity of pPI3K, pAkt, and pERK relative to GAPDH was determined by densitometric analysis and is indicated above each lane.

Internalization of ligand*-*activated receptors can initiate many cellular signaling events [4]. Different domains of the RVA surface proteins interact with different cell surface molecules including sialic acid (SA) or histo*-*blood group antigens (HBGAs), integrins and a heat shock cognate (hsc70) protein [5, 33–37]. Since the PI3K/Akt and MEK/ERK pathways were found to be activated during immediate early RVA infection, interaction of RVA surface proteins with its cellular receptors and/or coreceptors could trigger the activation of these cascades. To address which surface viral protein(s) could induce the activation of both signaling pathways, we used purified VP8*, VP5*, and VP7 of the RVA strains DS-1 and NCDV. Addition of 10 μg/ml of VP8*, VP5*, and VP7 proteins resulted in phosphorylation of PI3K and Akt as early as 2 min post-treatment, becoming obvious at 5 min post-treatment (Fig 9). Moreover, phosphorylation of ERK was detected 5 min after treatment with VP8*, VP5* and VP7 of either strain (Fig 9). However, none of the recombinant VP8*, VP5*, or VP7 proteins from the E-P RVA strain RRV could induce the activation of either of the two signaling cascades (S9 Fig), supporting that neither of the two signaling pathways are involved in entry of the RRV strain. To further confirm the above results, MA104 cells were pretreated for 1 h at 37°C with either wortmannin or U0126, incubated with each surface domain of the two RVA strains for 5 min, and analyzed for phosphorylation of Akt and ERK by Western blot analysis. The expression levels of pAkt and pERK were found to be reduced following wortmannin and U0126 pretreatment, respectively (S10 Fig).

Sodium periodate (NaIO_4_) can remove the VP8*-binding cell surface carbohydrate moieties, terminal SAs, and HBGAs without altering proteins or membranes. In particular, SAs can be removed by pretreatment with 1 mM NaIO_4_, whereas neutral glycan structures such as HBGAs can be eliminated by pretreatment with 10 mM NaIO_4_ [38, 39]. Therefore, we pretreated MA104 cells with 1 mM NaIO_4_ for the SA-dependent NCDV strain, and with 10 mM NaIO_4_ for the HBGA-dependent DS-1 strain. Removal of SAs and HBGAs inhibited both signaling pathways (S11A Fig). We next examined whether VP5*- and VP7-induced early activation of both signaling pathways could be reduced by pre-incubation with antibodies specific for the αVβ3 integrin at the VP7 CNP site [34, 36], and for Hsc70 at the VP5* KID site [40]. The results showed that depletion of the cellular receptors reduced VP5*- and VP7-induced early activation of both signaling pathways (S11B and S11C Fig). These data further support that the RVA outer capsid proteins VP4-VP8* and VP4-VP5* domains as well as VP7 can induce phosphorylation of signaling molecules PI3K, Akt, and ERK during entry of L-P RVA strains.

**Fig 10:**
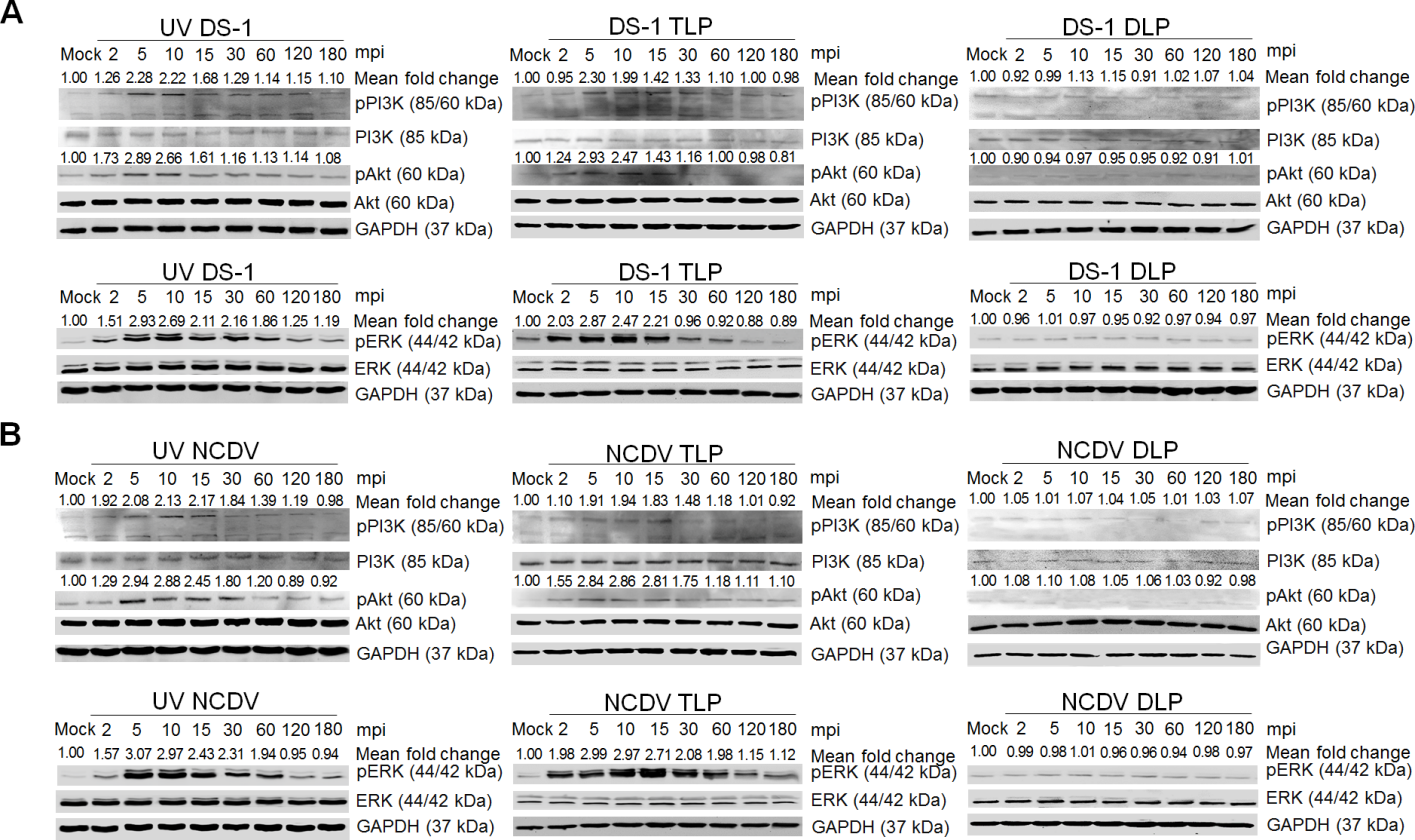
PI3K/Akt and MEK/ERK signaling pathways were activated by psoralen-UV-inactivated RVA and CsCl-purified RVA TLPs but not by intracellularly transfected RVA DLPs at the immediate early stage. (A-D) MA104 cells were independently infected with psoralen-UV-inactivated DS-1 and NCDV strains, or CsCl-purified RVA TLPs of DS-1 and NCDV strains, or transfected with RVA DLPs of DS-1 and NCDV strains, in a time dependent manner. Cell lysates were collected for Western blot analysis for the detection of PI3K, pPI3K, Akt, pAkt, ERK, and pERK. GAPDH was also analyzed and used as a loading control. The intensity of pPI3K, pAkt, and pERK relative to GAPDH was determined by densitometric analysis and is indicated above each lane.

To ensure that induction of these signaling molecules was indeed triggered by the outer capsid proteins but not by trypsin, pro-inflammatory cytokines, and/or other contaminants from the virus preparation, we first treated MA104 cells with 10 μg crystal trypsin and analyzed the activation of both signaling pathways. No significant changes were observed in the expression levels of pPI3K, pAkt, or pERK in mock- or crystal trypsin-treated cells (S12 Fig). We next infected the cells with the two purified 4’-aminomethyltrioxsalen hydrochloride (psoralen)-UV-inactivated strains, or with cesium chloride (CsCl)-purified TLPs of the two strains, or we transfected the cells with DLPs of the two strains. As shown in Fig 10, MA104 cells infected with psoralen-UV-inactivated strains or CsCl-purified TLPs of the two strains exhibited activation of pPI3K, pAkt, and pERK during the immediate phase. In contrast, these signaling molecules could not be activated in RVA DLPs-lipofected cells (Fig 10). Moreover, recombinant NSP1 and VP6 proteins of DS-1 and NCDV strains were used to rule out effects of other RVA proteins. No activation of the PI3K/Akt and MEK/ERK signaling pathways was seen in NSP1- or VP6-incubated MA104 cells (S13 Fig). Taken together, our results indicate that sequential multistep interactions of the outer capsid proteins of both L-P strains with their corresponding cellular receptors activate the PI3K/Akt and MEK/ERK signaling pathways.

## Discussion

Host defense systems have developed to eliminate invading viruses at different stages of virus infection [41, 42]. However, viruses, in turn, have evolved numerous strategies to disarm the host antiviral responses including evasion of host innate and adaptive immunities, and hijacking of the host cell machinery including a variety of host cell signaling pathways that modulate the intracellular environment at different stages of their life cycle [41, 42]. In addition to the strategies of RVA to antagonize the host innate immunity via the viral NSP1 and VP3 [28–30, 43–46], RVA also hijacks and alters host cellular signaling pathways during its life cycle [26, 27, 31, 41]. Among them, the PI3K/Akt and MEK/ERK signaling pathways have attracted much interest due to their multifaceted roles modulating virus entry, replication, assembly, and release [18–21]. Here, we demonstrate that the direct interaction of phosphorylated PI3K, Akt, and ERK molecules with the subunit E of the V-ATPase V_1_ domain in response to early infection of L-P RVA strains stimulates endosomal acidification, which is required for uncoating of TLP and delivering of DLP into the cytoplasm to continue the virus life cycle. Our findings extend our current understanding of the mechanisms of RVA entry and uncoating, and could contribute to devising useful new strategies for developing anti-RVSs drugs for treatment of the RVA infection.

L-P RVA strains such as the human strains Wa and DS-1, the porcine strain TFR-1, and the bovine strain UK are known to require late endosomal acidification for TLP uncoating and delivery of DLP into cytoplasm [4, 7, 10]. In agreement with the results of a recent report [10], we found that the human strain DS-1 and the bovine strain NCDV used in this study were L-P strains that required LE acidification. Both strains colocalized with the LE marker LAMP2 and their entry and infectivity depended on Rab7 as well as on endosomal acidification. However, to date, the molecular mechanism(s) driving LE acidification for uncoating of L-P strains remains largely unknown. The V-ATPase, with energy harnessed from ATP hydrolysis by phosphotransferases such as PI3K, Akt, and ERK targeted in this study, is known to translocate protons from the cytoplasmic to the luminal side of the membrane, resulting in luminal acidification including that of LE [15]. In the present study, the PI3K/Akt and MEK/ERK cascades were activated during the immediate early infection of both neuraminidase (NA)-sensitive DS-1 and NA-insensitive NCDV strains. Moreover, co-immunoprecipitation experiments using antibodies specific for pPI3K, pAkt, pERK, and the subunit E of the V_1_ domain of the V-ATPase, immunoprecipitated the V-ATPase or its counter partners, the signaling molecules pPI3K, pAkt, and pERK. Using the Duolink technology, we further proved that the subunit E of the V_1_ domain of the V-ATPase directly interacts with the signaling molecules pPI3K, pAkt, and pERK following RVA infection. Our data indicate that pPI3K, pAkt, and pERK induced during the immediate early infection of the L-P strains used in this study directly interact with V-ATPase to produce a proton gradient by ATP hydrolysis. These results are also supported by the finding that acidic replenishment of the medium restored uncoating of the L-P strains in cells pretreated with inhibitors specific for the PI3K/Akt and MEK/ERK signaling pathways. Our results are partially consistent with findings that influenza A virus-induced early activation of PI3K and ERK mediates V-ATPase-dependent endosomal acidification [25].

The PI3K/Akt and/or MEK/ERK signaling pathways are involved in entry of many viruses [22–24]. However, the interaction of host cell signaling pathways with L-P RVA strains for continuing their journey to the LE remains elusive. In this study, the L-P RVA strains DS-1 and NCDV could not be detected by antibody against the VP8* domain in the LEs at 120 mpi, suggesting that uncoating of these strains had been completed at this time point. The PI3K/Akt and MEK/ERK pathways were activated by these strains as early as 5 mpi. Moreover, inhibition of these signaling pathways by pretreatment with PI3K (wortmannin) or MEK (U0126) inhibitors markedly reduced infectivity, suggesting a possible involvement of these signaling pathways in entry of L-P strains. However, inhibition of both signaling pathways did not trap the incoming virus particles in the EEs. Instead, both L-P strains were retained in the LEs for as long as 6 hpi by these inhibitors. These data demonstrate that neither of the two signaling pathways is involved in trafficking of L-P RVAs, such as the DS-1 and NCDV strains, from the cell surface to LEs.

Binding of viral ligand(s) to host cell surface receptor(s) can activate signaling pathways through the plasma membrane, promoting virus uptake or penetration into the cells [1]. RVAs initiate the infection by a complex multistep process in which the VP8* and VP5*domains of the VP4 capsid protein, as well as the VP7 capsid protein interact with different cell surface receptors [4, 5]. Here, we demonstrate that the VP4-VP8* and VP4-VP5* domains, and the VP7 transiently activate the PI3K/Akt and MEK/ERK pathways during entry of the L-P strains DS-1 and NCDV. Inhibitors specific for each signaling pathway blocked the expression of both signaling pathways activated by the outer capsid proteins, further confirming that the outer capsid proteins of RVAs can activate both the PI3K/Akt and the MEK/ERK signaling pathways. This possibility was bolstered by the observation that psoralen-UV-irradiated RVAs, which were noninfectious but preserved the structural and immunological functions [47, 48], as well as CsCl-purified RVA TLPs, but not DLPs lipofection or trypsin treatment, could also activate the PI3K/Akt and MEK/ERK pathways. The immediate early activation of the PI3K/Akt and MEK/ERK pathways by DS-1 and NCDV was unlikely due to other proteins since the NSP1 and VP6 proteins failed to induce phosphorylation of PI3K, Akt, or ERK during the time of RVA entry. Our data indicate that the outer capsid proteins of RVAs, VP4-VP8* and VP4-VP5* domains, and VP7, can induce phosphorylation of PI3K, Akt, and ERK signaling molecules during the entry phase of L-P RVA strains, which in turn directly interact with the subunit E of the V_1_ domain of the V*-*ATPase to produce a proton gradient by ATP hydrolysis and subsequently acidify the LE for uncoating of RVAs.

There have been a considerable number of reports showing that activation of the PI3K/Akt and MEK/ERK signaling pathways can occur at multiple steps in the virus life cycle [18–21]. Activation of the PI3K/Akt signaling cascade has also been reported at different time points during the RVA life cycle [26–31]. Among these reports, Halasz and colleagues reported an RVA-induced sequential activation of the Akt signaling molecule in RVA-infected cells [26]. PI3K-dependent Akt phosphorylation was observed at 1 hpi, and it was sustained until 3 to 4 hpi in simian RRV strain-infected human intestinal epithelial Caco-2 and HT-29 cells, as well as in monkey kidney epithelial MA104 cells [26]. As confirmed in this study, the phosphorylated form of Akt molecule could not be detected at earlier time points during infection of RRV strain [26]. The simian RVA strain RRV does not require a deep journey to LEs since it is an E-P strain [12]. In addition, acidification of the EEs is not necessary for uncoating of this E-P strain [4, 11, 14]. These biological properties of E-P strains could make them be released DLP from EEs in to cytosol within 10 mpi [49]. Therefore, unlike L-P strains such as the DS-1 and NCDV strains used in this study, RRV strain does not need immediate early-activation of both PI3K/Akt and MEK/ERK signaling pathways for uncoating in acidified LEs. Rather, simian RVA RRV-induced early activation of the PI3K/Akt signaling pathway occurring from 1 hpi to 3–4 hpi, coinciding with the period for RRV-induced cell adhesion, plays a key role in the increased yield of infectious RRV particles by increasing the adhesion and survival of infected cells [26].

Following findings that RVA-induced early apoptosis in MA104 and HT29 cells was protected by RVA activation of PI3K [30], it becomes clear that the direct interaction between the RVA NSP1 protein and the p85 subunit of PI3K suppresses RVA-induced cellular apoptosis to facilitate viral growth [28, 29]. This interaction, in turn, activates the PI3K-dependent Akt signaling molecule from 2 to 12 hpi [28]. Hsp90, a molecular chaperone primarily involved in protein folding during protein synthesis, increases RVA replication through upregulation of the PI3K-dependent Akt signaling molecule during RVA protein synthesis, specifically from 4 to 12 hpi for the SA11 strain and from 2 to 6 hpi for the KU strain [27]. Hsp90 is also known to contribute to viral replication either by modulating cellular signaling pathways or by direct interactions with viral proteins such as the RNA dependent RNA polymerase of influenza A and vesicular stomatitis viruses, the reverse transcriptase of hepatitis B virus, and NSP2/3 protein of hepatitis C virus [27, 50–55]. Similarly, we observed a second activation phase of these signaling molecules from 4 to 12 hpi in both MA104 and Caco-2 cells, which could be triggered during and/or after RVA protein synthesis. RVA-induced immediate early activation of the PI3K and/or Akt signaling molecules observed in cells infected with the L-P strains DS-1 and NCDV used in the present work had not been examined in the previous studies [27–30]. Since activation of the PI3K-dependent Akt molecule observed in the previous studies seems to occur during and/or after RVA protein synthesis, including synthesis of the NSP1 protein, the molecular mechanism of PI3K/Akt signaling pathway activation observed during entry of RVAs in the present study could be distinct from that observed in RVA-induced early apoptosis and RVA protein synthesis [27–30].

**Fig 11:**
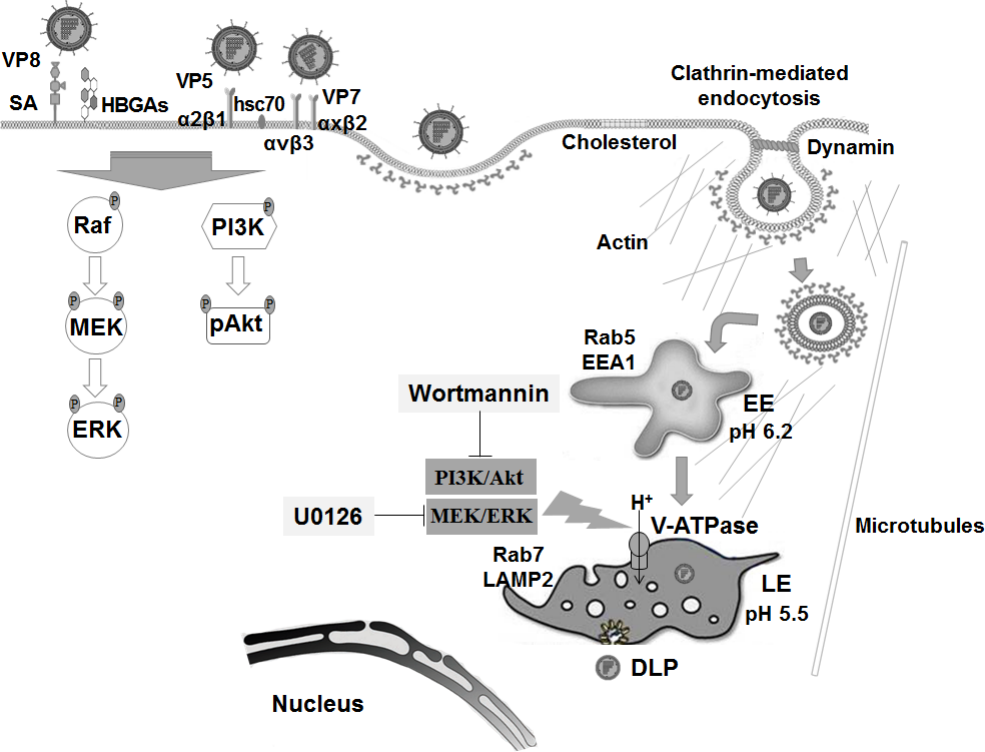
Schematic diagram for endosomal acidification by RVA-induced early activation of PI3K, Akt, and ERK signaling molecules. RVAs enter the cells by sequential multistep binding of outer capsid proteins (VP4-VP8*, VP4-VP5*, and VP7) to their cognate host cellular receptors and coreceptors, represented by SA, HBGAs, integrins, and Hsc70 protein, depending on the virus strain. Subsequently, RVA particles are internalized by clathrin-dependent or -independent endocytic pathways, depending on the virus strain. All RVA strains move to EEs expressing Rab5 and EEA1. In addition, some RVA strains travel to LEs (L-P viruses). Multistep binding of RVA outer capsid proteins (VP4-VP8*, VP4-VP5*, and VP7) to host cell surface receptors and coreceptors activates the PI3K/Akt and MEK/ERK signaling pathways. The phosphorylated signaling molecules, pPI3K, pAkt, and pERK, interact directly with the subunit E of the V_1_ domain of the V-ATPase to produce a proton gradient by ATP hydrolysis to acidify the LE for uncoating of RVAs.

In summary, this study demonstrates that the L-P RVA strains DS-1 and NCDV phosphorylate the signaling molecules PI3K, Akt, and ERK which, in turn, directly interact with the subunit E of the V_1_ domain of the V*-*ATPase to produce a proton gradient by ATP hydrolysis and subsequently acidify the LE for uncoating of RVAs (Fig 11). Activation of the signaling molecules PI3K, Akt, and ERK seems to be induced by binding of outer capsid proteins, VP4-VP8* and VP4-VP5* domains, and VP7, to known cell surface receptors. However, the PI3K/Akt and MEK/ERK signaling pathways induced during the immediate early infection of L-P strains were not involved in virus trafficking from the EEs to the LEs. This study provides a better understanding of the RVA*-*host interaction at the cell signaling level, which is of fundamental importance for developing strategies for the control and prevention of RVA infections.

## Materials and methods

### Cells, viruses, and infection

Monkey kidney MA104 cells obtained from the American Type Culture Collection (ATCC, Manassas, VA, USA) were grown in alpha minimal essential medium (α*-*MEM*)* (Welgene, Daegu, South Korea) supplemented with 10% fetal bovine serum (FBS), 100 U/ml penicillin, and 100 μg/ml streptomycin. Human intestinal Caco-2 cells (ATCC) were grown in Dulbecco's modified Eagle's medium (DMEM) supplemented with 10% FBS, 100 U/ml penicillin, and 100 μg/ml streptomycin. *Spodoptera frugiperda* ovarian cells (Sf9 cells) purchased from Gibco (Fort Worth, Texas, USA) were cultured at 27°C in SF*-*900 II SFM media containing 10% FBS, 100 U/ml penicillin, 100 μg/ml streptomycin, lipid medium supplement, and 0.1% pluronic acid solution (Sigma Aldrich, St. Louis, MO, USA).

The human RVA DS-1 (G2P1B[4]) and bovine RVA NCDV (G6P6[1]) strains were purchased from the ATCC, and the rhesus RVA strain RRV was kindly provided by Professor Susana López (Instituto de Biotecnología, Cuernavaca, Morelos, México). Both strains were preactivated with 10 μg/ml crystalized trypsin (Cat. No. 27250*–*018, Gibco), and propagated in MA104 cells as previously described [56]. RVA TLPs and DLPs were purified by CsCl isopycnic gradients as previously described [57]. Virus titers were determined by cell culture immunofluorescence (IF) assay using monoclonal antibodies (Mabs) specific for RVA VP6, and are expressed as fluorescence focus units per milliliter (FFU/ml).

### Reagents and antibodies

Chloroquine, NH_4_Cl, psoralen and NaIO_4_ were purchased from Sigma-Aldrich. Wortmannin (PI3K inhibitor) and U0126 (MEK inhibitor) were obtained from Invivogen (San Diego, CA, USA). AF594 succinimidyl ester was purchased from Molecular Probes (Bedford, MA, USA). Wortmannin, U0126, and AF594 were dissolved in DMSO, while chloroquine, NH_4_Cl, and psoralen were dissolved in distilled water to prepare stock solutions. Before each use on cell monolayers, these chemicals were freshly diluted to the desired concentration with free media. The cytotoxic effects of the chemicals and their solvents were tested using the 3*-*(4, 5*-*dimethylthiazol*-*2*-*yl)*-*2,5*-*diphenyltetrazolium bromide (MTT) assay as previously described [38]. All the chemicals were used at concentrations that were not toxic to the cells. AF488-labeled phalloidin, cell tracker green 5*-*chloromethylfluorescein diacetate (CMFDA), and SlowFade Gold antifade reagent with 4’,6*-*diamidino*-*2*-*phenylindole (DAPI) were obtained from Molecular Probes. Protein A*-*agarose and protein G plus*-*agarose were purchased from Santa Cruz (Dallas, Texas, USA). All the siRNAs were purchased from Santa Cruz and the sequences are presented in S2 Table.

Specific rabbit polyclonal antibodies against Akt, pAkt at the active site serine 473, PI3K p85 regulatory subunit, pPI3K at the p85 regulatory subunit tyrosine 458 and at the p55 regulatory subunit tyrosine 199, Rab5, ERK1/2, and pERK1/2 at the threonine 202 and tyrosine 204 were purchased from Cell Signaling (Beverly, Massachusetts, USA). Goat anti*-*V*-*ATPase E subunit (ATP6E) and rabbit anti-glyceraldehyde 3-phosphate dehydrogenase (GAPDH, FL*-*335) polyclonal antibodies were from Santa Cruz. Mouse monoclonal and rabbit polyclonal antibodies against Na^+^/K^+^-ATPase B1 protein were purchased from Santa Cruz. Rabbit anti-Hsc70 polyclonal antibody was obtained from GeneTex (Irvine, CA, USA) and mouse anti-αVβ3 Mab was from Millipore (Temecula, CA, USA). Rabbit anti*-*Rab7 and mouse LAMP2 Mabs were purchased from Abcam (Cambridge, MA, USA). Mouse anti*-*EEA1 Mab was obtained from BD Transduction Laboratories (Lexington, KY, USA). Mouse anti*-*RVA VP6 Mab was purchased from Median Diagnostic (Chuncheon, South Korea), and hyperimmune rabbit sera raised against the VP8* domain of the DS*-*1 and NCDV strains were produced in this study. Secondary antibodies included horseradish peroxidase (HRP)*-*conjugated goat anti*-*rabbit IgG (Cell Signaling), HRP*-*conjugated goat anti*-*mouse IgG (Ab Frontier, Seoul, South Korea), HRP*-*conjugated donkey anti*-*goat IgG, fluorescein isothiocyanate (FITC)*-*conjugated anti-rabbit IgG, FITC*-*conjugated anti*-*mouse IgG (Santa Cruz), AF594*-*conjugated donkey anti*-*rabbit IgG, AF647*-*conjugated goat anti*-*rabbit IgG, AF594-conjugated goat anti-mouse IgG, and AF647-conjugated goat anti*-*mouse IgG (Life Technologies, Eugene, OR, USA).

### Labeling of viruses with AF594 and virus quantitation by TEM

AF594*-*labeling of DS-1 or NCDV strains purified by CsCl density-gradient centrifugation was performed as previously described [58]. Briefly, purified RVA TLPs (10 mg at 1 mg ml^-1^) in 0.1 M sodium bicarbonate buffer (pH 8.3) was labeled with one-tenth-fold-molar concentration of AF594 succinimidyl ester (1 mg at 1 mg ml^-1^ in DMSO. Each reaction was mixed thoroughly by vortexing for 30 s and incubated for 1 h at room temperature with continuous stirring. This fluorophore reacts exclusively with free amines, resulting in a stable carboxamide bond, and contains a seven*-*atom aminohexanoyl spacer (X), which allows higher degree of labeling without functional perturbance of the virus [58]. The labeled virus was repurified by CsCl density-gradient centrifugation, dialysed against virion buffer, and stored in 2 μg aliquots at -20°C [58]. Analysis of sodium dodecyl sulfate*-*polyacrylamide gel (SDS*-*PAGE)-separated AF594 labeled viral particles by Coomassie blue staining and Western blotting showed that the label was exclusively coupled to the viral protein.

The number of RVA particles was examined as described previously [59, 60]. Briefly, CsCl-purified AF594-RVA particles were mixed with an equal volume of a suspension of 120 nm latex beads (Sigma Aldrich). The mixture was then applied to the grids. The grids were stained with 3% phosphotungstic acid (PTA) at pH 7, for 3 min, at room temperature and observed under a JEM-2100F transmission electron microscope (Jeol, Peabody, MA, USA). The virus particles were counted along with the beads in at least 10 randomly chosen squares on the grid. The total virus count was calculated by multiplying the ratio of the virus particle number to the latex particle number by the known latex particle concentration per ml.

### Psoralen inactivation of RVAs

Genetic inactivation of cell culture-derived RVAs was performed using psoralen and long-wave UV light exposure as described elsewhere [47, 48]. Briefly, 2 ml of virus suspension was mixed with psoralen at 20 μg/ml in a petri dish and incubated at 4°C for 15 min. Virus was then exposed to UV light at 366 nm for 40 min. The effectiveness of psoralen-UV inactivation was demonstrated by the lack of detectable viral antigen in an immunofluorescence assay in MA104 cells infected with psoralen-UV-treated RVAs.

### Pretreatment of cells with inhibitory chemicals or antibodies

MA104 or Caco-2 cells were grown in 6- or 12*-*well plates or in 8-well chamber slides to the desired confluency, washed twice with phosphate-buffered saline (PBS, pH 7.4), and mock-treated or pretreated with working concentrations of the chemicals for 1 h at 37°C. Treatment with NaIO_4_ was conducted for 30 min at 4°C [38, 39], and antibodies were incubated for 2 h at 37°C [34]. Inhibitory chemicals and antibodies were used at the following concentrations: chloroquine (100 μM), NH_4_Cl (100 mM), wortmannin (10 nM and 1 μM), U0126 (0.25 μM and 50 μM), NaIO_4_ (1 mM and 10 mM), rabbit anti-Hsc70 polyclonal antibody (10 μg/ml), and anti-αVβ3 Mab (10 μg/ml). After washing the cells twice with PBS, the cells were infected with AF594*-*labeled or mock-labeled virus or treated with each purified viral protein independently, and then used for assessing signaling pathways and for measuring virus infectivity and titer by IF assay, genome copy number by RT*-*qPCR, and protein expression by Western blot as described below.

### Transfection of siRNA and DLPs

MA104 or Caco-2 cells cultured in 8*-*well chamber slides or 12*-*well culture plates to 70*–*80% confluency were transfected with siRNA (80 pmol of scrambled control siRNA and siRNAs against Rab5 and Rab7, or 40 nmol of siRNAs against PI3K p85α and MEK) using the Lipofectamine 2000 (Invitrogen, Carlsbad, California, USA) following the manufacturer’s instructions. To optimize knockdown efficiency, a second transfection was carried out 24 h after the first transfection. Cells treated in parallel were evaluated by Western blot analysis to ensure effective knockdown of each target protein. After confirming knockdown of each target protein, the cells were infected with AF594*-*labeled or mock*-*labeled virus and then used for assessing signaling pathways and for measuring virus infectivity and titer by IF assay, genome copy number by RT*-*qPCR, and protein expression by Western blot analysis as described below. Transfection of DLPs was performed using Lipofectamine 2000 (Invitrogen) as described previously [46]. Briefly, DLPs (10 μg/ml) were diluted in opti-MEM and incubated with a mixture of Lipofectamine in opti-MEM for 20 min at room temperature. One hundred microliters of this mixture was added to the cells for 1 h at 37°C. After removal of the lipofection mixture, the medium was replaced with MEM and cells were incubated for the indicated times.

### Cloning, expression and purification of RVA VP8*, VP5* and VP7 proteins

Recombinant VP8* domain of the RVA strains DS*-*1, NCDV and RRV were cloned, expressed and purified as described previously [37]. Briefly, the cDNAs encoding the VP8* domain with a cysteine peptide were cloned into the expression vector pGEX*-*4T*-*1 (glutathione S*-*transferase [GST]-gene fusion system) (GE Healthcare Life Sciences, Piscataway, NJ, USA). After sequence confirmation, the recombinant GST*-*VP8* fusion proteins were expressed in *Escherichia coli* (*E*. *coli*) strain BL21. Expression of each domain was induced with isopropyl*-*β*-*D*-*thiogalactopyranoside (IPTG; 0.2 mM) at room temperature overnight. RVA GST*-*VP8* fusion proteins were purified using the Pierce GST spin purification kit (Pierce, IL, USA) according to the manufacturer’s protocol. Recombinant RVA NSP1 and VP6 proteins were also expressed and purified as mentioned before [61–62]. The full genome amplicons of NSP1 and VP6 were cloned into plasmids pET28a and pPROEX HTc, respectively, digested with NcoI and XhoI, and the resulting constructs were verified by sequencing. The NSP1 and VP6-containing plasmids were expressed in *E*. *coli* BL21. Protein expression was induced with IPTG (1 mM) at room temperature overnight. The His-tagged NSP1 and VP6 proteins were purified using Ni*-*NTA agarose (Qiagen, Valencia, CA, USA), and the proteins were eluted with 500 mM imidazole. The concentration of the purified RVA VP8* domain, and NSP1 and VP6 proteins was determined by measuring the absorbance at 280 nm.

Recombinant VP5* and VP7 proteins are usually insoluble, difficult to express and purify, or toxic to the cell if expressed in *E*. *coli* [35, 63–66]. Since baculovirus has been successfully used as an expression system for the production of RVA proteins in the past [66–70], we expressed recombinant VP5* and VP7 proteins in baculovirus*-*infected Sf9 cells using the Bac*-*to*-*Bac Baculovirus expression system (Invitrogen) according to the manufacturer’s instructions. Briefly, the complete sequence of the VP5* domain and the VP7 gene of DS*-*1, NCDV, and RRV strains were amplified by RT*-*PCR with primers specific for each domain or gene (S1 Table) [66, 69]. Subsequently, the amplified fragments tagged with polyhistidine were subcloned into the donor plasmid pFastBac1. Recombinant baculovirus was generated by transformation of the recombinant pFastBac1 plasmid into DH10Bac *E*. *coli* to produce recombinant Bacmid DNA, which was then transfected into Sf9 cells using Cellfectin II reagent (Invitrogen). Recombinant VP5* and VP7 were expressed in baculovirus*-*transformed Sf9 insect cells at 27°C and harvested 5*–*7 days post*-*infection. After clarification by centrifugation, the polyhistidine*-*tagged proteins were purified using Ni*-*NTA agarose (Qiagen, Valencia, CA, USA) according to the manufacturer’s protocol. The concentration of purified RVA VP5* domain and VP7 protein was determined by measuring the absorbance at 280 nm. Each purified protein was used at 10 μg/ml to test whether they could activate target signaling pathways by Western blot analysis as described below [35].

### Virus internalization assay

Subconfluent MA104 cells grown in 8*-*well chamber slides, pretreated with or without the chemicals of interest or siRNA, were washed twice with PBS. Then, AF594*-*labeled DS-1 (595 particles/cell) or NCDV (790 particles/cell) were allowed to bind to the cells for 30 min at 4°C, followed by incubation at 37°C to allow entry. Cells were washed extensively with cold PBS, and fixed with 4% paraformaldehyde in PBS for 15 min at room temperature. For colocalization with early and late endosomes markers, mock-, chemical- or siRNA-treated MA104 cells were independently infected with the RVA strains DS-1 and NCDV. The cells were then fixed with 4% paraformaldehyde in PBS for 15 min at room temperature, and permeabilized by addition of 0.2% Triton X*-*100 in PBS for 10 min at room temperature. Next, the cells were washed with PBS containing 0.1% new born calf serum (PBS*-*NCS) and incubated at 4°C overnight with Mabs against EEA1 and LAMP2, and polyclonal antibody against RVA VP8* domain (1:100 dilution), or Mabs against Rab5 and Rab7 (1:100 dilution). After washing twice with PBS-NCS, the cells were incubated with AF647*-*conjugated goat anti*-*mouse (1:100 dilution) or anti-rabbit IgG (1:100 dilution) antibodies for 1 h at room temperature. Immediately after washing with PBS-NCS, the cells were incubated with AF488*-*labeled phalloidin (10 units) (Invitrogen) for 15 min at room temperature for cytoskeleton staining and washed with PBS. Finally, chambers were mounted with SlowFade Gold antifade reagent containing 1 x DAPI solution (Molecular Probes) for nucleus staining, and the infected cells were observed with a LSM 510 confocal microscope and analyzed using the LSM software (Carl Zeiss).

Low*-*pH rescue experiment was performed as described previously [11]. Briefly, infected cells were incubated with either neutral (pH 7.2) or acidic (pH 5) citrate buffers for 5 min at 37°C. After washing three times with PBS, the cells were incubated in serum*-*free medium for 2 h at 37°C. Subsequently, the cells were fixed, permeabilized and stained with anti*-*RVA VP8* and anti*-*LAMP2 antibodies as described above.

### Determination of virus infectivity, genome copy number, and protein expression

To determine virus infectivity, genome copy number, and protein expression, confluent monolayers of MA104 cells grown in 6*-* or 12*-*well plates or 8*-*well chamber slides were pretreated with or without various inhibitors or transfected with or without siRNAs as described above. MA104 cells were then independently infected with trypsin-preactivated DS*-*1 and NCDV strains (10 μg/ml crystalized trypsin) at a MOI of 10 FFU/cell. Virus inocula were removed after 1 h of infection, and cells washed twice with PBS were used for determining the virus infectivity and titer by IF assay, genome copy number by RT-qPCR, and protein expression by Western blot analysis as described below.

### IF assay for the determination of virus infectivity and titer

To determine virus infectivity after pretreatment with chemicals or siRNAs, an IF assay was performed as described previously with minor modifications [38]. Briefly, MA104 cells grown in 8*-*well chamber slides, pretreated with or without chemicals or transfected with or without siRNAs were infected with trypsin-preactivated DS*-*1 and NCDV strains (10 μg/ml crystalized trypsin) at a MOI of 10 FFU/cell. Virus inocula were removed after 1 h of infection, and cells were washed twice with PBS. Cells were incubated for 8 h with medium containing 1 μg/ml crystalized trypsin, fixed with 4% paraformaldehyde for 15 min at room temperature, and permeabilized by addition of 0.2% Triton X*-*100 for 10 min at room temperature before being washed with PBS. The chamber slides were then incubated with Mabs against the RVA VP6 protein at 4°C overnight. Subsequently, cells were washed 3 times with PBS, and FITC*-*conjugated secondary antibodies were added. After washing with PBS*-*NCS, the nuclei were stained with DAPI, and cells were examined by confocal microscopy. Infected cells and total DAPI*-*stained cells were counted, and were scored for RVA VP6 expression. After image analysis with Zeiss LSM image browser (Oberkochen, Germany), infected cells were counted as positive for viral antigen if they had a fluorescence intensity at least three times that of the uninfected controls. The percentage of positive cells was then normalized to that of the untreated control.

To determine the RVA titer after pretreatment with chemicals or siRNAs, the IF assay was performed. Briefly, MA104 cells grown in 8*-*well chamber slides pretreated with or without chemicals or transfected with or without siRNA were independently infected with trypsin-preactivated DS*-*1 and NCDV strains (10 μg/ml crystalized trypsin) for 8 h. After three times of freeze*-*thaw cycles, a ten*-*fold dilution of each sample was used to infect in triplicate confluent MA104 cells grown in 96*-*well plates. After an adsorption period of 1 h at 37°C, the virus inocula were removed and the cells were washed with PBS. Subsequently, the infection was continued at 37°C for 16 h with medium containing 1 μg/ml crystalized trypsin. The cells were fixed with 80% cold acetone. After washing with PBS (pH 7.4), each well was incubated with Mab against the RVA VP6 protein at 4°C overnight. Subsequently, cells were washed 3 times with PBS, and FITC*-*conjugated secondary antibodies were added. After washing with PBS (pH 8.0*)*, the nuclei were stained with DAPI. The viral titer was expressed as FFU/ml.

### Western blot analysis

To assess the expression levels of each viral protein and target cellular protein under the conditions described above, antibodies specific for each protein were used for Western blot analysis. Briefly, MA104 cells grown in 6- or 12*-*well plates and treated with factors including chemicals, siRNAs, and viruses, were washed three times with cold PBS and lysed using cell extraction buffer containing 10 mM Tris/HCl pH 7.4, 100 mM NaCl, 1 mM EDTA, 1 mM EGTA, 1 mM NaF, 20 mM Na_2_P_2_O_7_, 2 mM Na_3_VO_4_, 1% Triton X*-*100, 10% glycerol, 0.1% SDS, and 0.5% deoxycholate (Invitrogen) for 30 min on ice. To determine signaling molecules induced by recombinant VP8* and VP5* domains as well as recombinant VP7 protein, GST*-*VP8*, His*-*VP5*, and His*-*VP7 (10 μg/ml) were independently incubated with confluent MA104 cells grown in 6*-*well plates for 30 min at 4°C, shifted to 37°C for the indicated times, washed, and lysed as described above. Lysates were spun down by centrifugation at 12,000×g for 10 min at 4°C and the supernatants were analyzed for total protein content with a BCA protein assay kit (Thermo Scientific, Waltham, MA, USA). Samples were resolved by SDS*-*PAGE and transferred onto nitrocellulose membranes (GE Healthcare Life Sciences). The membranes were blocked for 1 h at room temperature with Tris*-*buffered saline containing 5% skimmed milk before they were incubated overnight at 4°C with the indicated primary antibodies. The bound antibodies were developed by incubation with a HRP*-*labeled secondary antibody and the immunoreactive bands were detected by enhanced chemiluminescence (ECL) (Dogen, Seoul, South Korea) using a Davinch*-*K Imaging System (Youngwha Scientific Co., Ltd, Seoul, South Korea).

### Immunoprecipitation

Immunoprecipitation of each target protein was performed as previously described [25]. Briefly, MA104 cells grown in 6*-*well plates were independently infected with DS*-*1 and NCDV strains at a MOI of 10 FFU/cell, or mock-infected, and incubated for the indicated time points at 37°C. Afterwards, the cells were washed and lysed as described above. Cell lysates were pre*-*cleared by incubation with protein A- or G-agarose beads for 30 min at 4°C. Subsequently, the pre*-*cleared cell lysates were incubated with antibodies against the V*-*ATPase E subunit, pPI3K, pAkt, and pERK, or with an irrelevant antibody against Na^+^/K^+^-ATPase B1 protein overnight at 4°C. The immune complexes were captured by incubation with A- or G- agarose beads for 1 h at 4°C, and the immunoprecipitated proteins were then evaluated by Western blot analysis as described above. To rule out entrapment of any of the V-ATPase, pPI3K, pAkt, or pERK during immunoprecipitation, the pre-cleared cell lysates described above were incubated with antibodies against the V-ATPase E subunit, pPI3K, pAkt, and pERK. Instead of capturing by incubation with A- or G-agarose beads, secondary antibodies specific for each primary antibody were added into each reaction mixture. The immune complexes were then captured by incubation with A- or G-agarose beads and the immunoprecipitated proteins were evaluated by Western blot analysis as described above.

### Duolink proximity ligation assay (DPLA)

*In situ* interactions of pPI3K, pAkt, and pERK with the V-ATPase were detected with the Duolink PLA kit (Sigma-Aldrich) as described elsewhere [32]. Briefly, RVA-infected MA104 cells grown in 8-well chamber slides were fixed with 4% paraformaldehyde in PBS for 15 min and permeabilized by addition of 0.2% Triton X*-*100 for 10 min at room temperature. The cells were then incubated with Duolink blocking solution in a pre-heated humidity chamber for 30 min at 37°C followed by incubation with primary antibodies, goat anti*-*V*-*ATPase E subunit, and rabbit anti-pPI3K, pAkt or pERK antibodies, or incubation with the control irrelevant rabbit or mouse antibodies against Na^+^/K^+^-ATPase B1 protein, and the above primary antibodies, overnight at 4°C. After washing twice in Duolink washing buffer A for 5 min, the cells were incubated with secondary antibodies conjugated with oligonucleotides (PLA probes anti-rabbit MINUS and anti-goat PLUS) for 1 h in a pre-heated humidity chamber at 37°C. Unbound PLA probes were removed by washing twice in Duolink washing buffer A for 5 min, and then the Duolink ligation solution was applied to the slides for 30 min in a pre-heated humidity chamber at 37°C followed by washing in Duolink washing buffer A twice for 2 min. The Duolink amplification-polymerase solution was applied to the slides in a dark pre-heated humidity chamber for 100 min at 37°C. The slides were then washed twice in 1x Duolink washing buffer B for 10 min followed by washing for 1 min with 0.01x Duolink washing buffer B. The cells were then mounted using Duolink *in situ* mounting medium with DAPI and observed with a LSM 510 confocal microscope (Carl Zeiss). PLA signals were recognized as red fluorescent spots.

### Real-time RT-PCR

To quantify the genome copy numbers of RVA, real*-*time RT*-*PCR was carried out as described previously with minor modifications [71]. MA104 cells grown in 12*-*well plates were pretreated with or without the indicated concentration of chemicals or transfected with or without siRNAs as described above. The cells were then infected with the strains DS-1 and NCDV at a MOI of 10 FFU/cell for 1 h. Next, the unbound viruses were removed by washing the cells with PBS. At 8 hpi, the infected cell cultures were washed twice with PBS, harvested by freezing and thawing three times, and cell debris was spun down at 2,469×g for 10 min at 4°C. The supernatants along with the remaining bulk samples were collected and stored at -80˚C until used. Total RNA was extracted using RNeasy kit (Qiagen) following the manufacturer’s instructions. The viral genome copy number was determined by one*-*step SYBR Green real*-*time RT*-*PCR using primer pairs specific for the DS*-*1 VP6 or NCDV VP6 genes (S1 Table). Each reaction mixture in a total volume of 20 μl contained 4 μl of RNA template (1μg), 10 μl SensiFast SYBR Lo*-*ROX One step mixture (Bioline, Quantace, London, UK), 0.8 μl each of 10 μM forward and reverse primers, 0.2 μl of reverse transcriptase, 0.4 μl of RiboSafe RNase inhibitor, and 3.8 μl of RNase*-*free water. Real*-*time RT*-*PCR was performed using a Rotor*-*Gene Real*-*Time Amplification system (Corbett Research, Mortlake, Australia) with the following conditions: reverse transcription was carried out at 50°C for 30 min, followed by activation of the hot*-*start DNA polymerase at 95°C for 10 min and 40 cycles of three steps of 95°C for 15 s, 50°C for 30 s, and 72°C for 20 s. Quantitation of viral RNA was carried out using a standard curve derived from serial 10*-*fold dilutions of complementary RNA (cRNA) generated by reverse transcription of *in vitro* transcribed control RNA (RVA VP6 gene). The threshold was automatically defined in the initial exponential phase, reflecting the highest amplification rate. A direct relationship between cycle number and the log concentration of RNA molecules initially present in the RT*-*qPCR reaction was used to calibrate the crossing points resulting from the amplification curves of the samples.

### Detection of acidic intracellular compartments

The pH probe cell tracker green CMFDA was used to visualize intracellular acidic compartments as described previously [72]. After chemical treatment and virus infection, MA104 cells were washed with PBS and incubated with cell tracker CMFDA working solution (10 μM) for 30 min at 37°C, which was then replaced with serum*-*free media and incubated for another 30 min at 37°C. Afterwards, fixation and permeabilization were performed as described above. To check colocalization with late endosome marker, the cells were incubated with anti*-*LAMP2 antibody and then examined by confocal microscope as described above.

### Statistical analyses and software

Statistical analyses were performed on triplicate experiments by One*-*Way ANOVA using GraphPad Prism software version 5.03 (GraphPad Software Inc., La Jolla, CA, USA). *P* values of less than 0.05 were considered statistically significant. Figures were generated using Adobe Photoshop CS6 and Prism 5 version 5.03.

## Supporting information

S1 Table Oligonucleotide primers used in this study. (DOCX)

S2 Table Sequences of siRNAs against target molecules and scrambled siRNA used in this study. (DOCX)

S1 Fig RVA-induced early activation of PI3K, Akt, and ERK signaling molecules in Caco-2 cells. (A and B) Caco-2 cells were mock-infected or infected with the DS-1 or NCDV strains (MOI = 10 FFU/cell) for the indicated time. The cells were then harvested at the indicated time points. The cell lysates were subjected to Western blot analysis to check the expression levels of phosphorylated PI3K (pPI3K), PI3K, pAkt, Akt, pERK, and ERK using the relevant antibody. GAPDH was used as a loading control. (C) Caco-2 cells were mock treated or pretreated with wortmannin or U0126 at the indicated doses for 1 h at 37°C, followed by infection with DS-1 and NCDV. Cell lysates were harvested at 5 mpi and the expression levels of pAkt, Akt, pERK, and ERK were evaluated by Western blot analysis using the relevant antibody. GAPDH was used as a loading control. (D) Caco-2 cells were transfected with scrambled siRNA or siRNAs specific for PI3K p85α or MEK, and then infected with either the human RVA DS-1 or the bovine RVA NCDV strains (MOI = 10 FFU/cell). The cell lysates were subjected to Western blot analysis to check the expression levels of pAkt, Akt, pERK, and ERK using the corresponding antibody. GAPDH was used as a loading control. The intensity of pPI3K, pAkt, and pERK relative to GAPDH was determined by densitometric analysis and is indicated above each lane.
(TIF)

S2 Fig The simian rotavirus RRV strain could not trigger immediate early activation of the PI3K, Akt, and ERK signaling molecules. MA104 cells (A) and Caco-2 cells (B) were infected with the simian RVA strain RRV (MOI = 10 FFU/cell) for the indicated time points. The cell lysates were subjected to Western blot analysis to check the expression levels of phosphorylated PI3K (pPI3K), PI3K, pAkt, Akt, pERK, and ERK using the relevant antibody. GAPDH was used as a loading control. The intensity of pPI3K, pAkt, and pERK relative to GAPDH was determined by densitometric analysis and is indicated above each lane.
(TIF)

S3 Fig Inhibition of the PI3K/Akt signaling cascade affects rotavirus infectivity and viral protein expression. MA104 cells were pretreated with a non*-*cytotoxic concentration of wortmannin for 1 h at 37°C and then infected with the RVA strains DS*-*1 and NCDV (MOI = 10 FFU/cell) for 8 h. The total viral RNA (A), antigen*-*positive cells (using anti*-*RVA VP6 Mab) (B and C), and VP6 protein (D) were determined by real*-*time RT*-*PCR, immunofluorescence, and Western blot analyses, respectively. GAPDH was used as a loading control. The intensity of pPI3K, pAkt, and pERK relative to GAPDH was determined by densitometric analysis and is indicated above each lane. (E) The virus titer was determined by cell culture immunofluorescence assay using cell lysates produced by 3 cycles of freezing and thawing; the results are expressed as fluorescent focus forming unit (FFU). All experiments were performed in triplicate; panel B shows a representative set of results. Data are presented as means ± standard error of the mean from three independent experiments. Differences were evaluated using the One-Way ANOVA. *p<0.05; **p<0.001; ***p<0.0001. The scale bars in panel B correspond to 20 μm.
(TIF)

S4 Fig Inhibition of the MEK/ERK signaling cascade affects rotavirus infectivity and viral protein expression. MA104 cells were pretreated with a non-cytotoxic concentration of U0126 for 1 h at 37°C and then infected with the RVA strains DS*-*1 and NCDV (MOI = 10 FFU/cell) for 8 h. The total viral RNA (A), antigen-positive cells (using anti*-*RVA VP6 Mab) (B and C), and VP6 protein (D) were determined by real*-*time RT*-*PCR, immunofluorescence, and Western blot analyses, respectively. GAPDH was used as a loading control. The intensity of pPI3K, pAkt, and pERK relative to GAPDH was determined by densitometric analysis and is indicated above each lane. (E) The virus titer was determined by cell culture immunofluorescence assay using cell lysates produced by 3 cycles of freezing and thawing and are expressed as FFU. All experiments were performed in triplicate and panel B shows a representative set of results. Data are presented as means ± standard error of the mean from three independent experiments. Differences were evaluated by the One-Way ANOVA. *p<0.05; **p<0.001; ***p<0.0001. The scale bars in panel B correspond to 20 μm.
(TIF)

S5 Fig Determination of chemicals cytotoxicity by MTT assay. (A-D) MA104 cells grown in 96-well plates were incubated with various concentrations of the indicated chemicals in triplicate for 24 h at 37°C. Afterwards, the chemicals-containing media was thoroughly removed and replaced with 200 μl of MTT solution for 4 h at 37°C. Each well was incubated with 100 μl of DMSO for 10 min at room temperature. Cell viability was measured using an ELISA reader at an OD value of 570 nm. The arrows indicate the concentrations used in this study.
(TIF)

S6 Fig Simian rotavirus RRV strain entry is not affected by inhibition of the PI3K/Akt and MEK/ERK signaling pathways. (A) MA104 cells were pretreated with or without chloroquine, wortmannin, or U0126 for 1 h at 37°C and subsequently infected with the RRV strain (MOI = 10 FFU/cell) for 30 min at 37°C. Cells were then incubated for 30 min with CMFDA (10 μM) to visualize acidification of intracellular compartments followed by 30 min in serum*-*free media. (B) MA104 cells were pretreated with or without wortmannin or U0126 for 1 h at 37°C and then infected with the RRV strain (MOI = 10 FFU/cell) for 3 h. After fixation and permeabilization, the cells were prepared for confocal microscopy using anti*-*TLP, anti*-*EEA1, and anti-LAMP2 antibodies, and the relevant secondary antibodies. Representative images are shown. The scale bars correspond to 10 μm (A) and 5 μm (B).
(TIF)

S7 Fig Confirmation of direct interaction of pPI3K, pAkt, and pERK with subunit E of the V-ATPase V_1_ domain for endosomal acidification. (A) Serum-starved MA104 cells were inoculated with RVA DS-1 or NCDV (MOI = 10 FFU/cell) for the indicating time. Subsequently, the cell lysates were immunoprecipitated using antibody specific for the Na^+^/K^+^-ATPase B1. (B) To rule out entrapment of any of the V*-*ATPase, pPI3K, pAkt, and pERK during immunoprecipitation, the pre-cleared cell lysates were incubated with antibodies against the V*-*ATPase, pPI3K, pAkt, and pERK. Each reaction mixture was then incubated with secondary antibodies specific for each primary antibody. The co-immunoprecipitated products and control inputs (C) were analyzed by Western blot analysis to detect pPI3K, pAkt, pERK, V_1_ subunit E, and Na^+^/K^+^-ATPase B1 using the relevant antibodies. GAPDH was used as a loading control.
(TIF)

S8 Fig The irrelevant Na^+^/K^+^-ATPase B1 protein did not interact with pPI3K, pAkt, pERK, or V-ATPase by Duolink proximity assay. Serum-starved MA104 cells were either mock-inoculated or inoculated with the RVA strains DS-1, NCDV, and RRV (MOI = 10 FFU/cell). Subsequently, the cells were fixed, permeabilized and incubated with two primary antibodies, the primary antibody against Na^+^/K^+^-ATPase B1 protein and primary V-ATPase E subunit, pPI3K, pAkt, or pERK antibodies overnight at 4°C. The Duolink PLA was performed as described in the Materials and Methods section and the signals are represented as red dots. Representative images are shown. The scale bars correspond to 20 μm.
(TIF)

S9 Fig Simian rotavirus RRV strain outer capsid proteins could not activate the PI3K/Akt and MEK/ERK signaling pathways. Serum*-*starved MA104 cells were incubated with the recombinant GST-fused VP8* (A) or his-tagged VP5* (B) or VP7 proteins (C) of the RRV strain at 10 μg/ml for the indicated time points. The cell lysates were subjected to Western blot analysis for the detection of pPI3K, pAkt, pERK, PI3K, Akt, and ERK using the relevant antibodies. GAPDH was used as a loading control. The fold change of pPI3K, pAkt, and pERK relative to GAPDH was determined by densitometric analysis and is indicated above each lane.
(TIF)

S10 Fig RVA outer capsid proteins-induced early activation of PI3K/Akt and MEK/ERK signaling pathways. MA104 cells were mock-treated or treated with wortmannin or U0126 and then incubated with purified VP8*, VP5*, or VP7 proteins of RVA DS*-*1 (A) and NCDV (B) strains at 10 μg/ml. Cell lysates were harvested at 5 mpi and the expression levels of pAkt, Akt, pERK, and ERK were evaluated by Western blot analysis. GAPDH was used as a loading control. The intensity of pPI3K, pAkt, and pERK relative to GAPDH were determined by densitometric analysis and is indicated above each lane.
(TIF)

S11 Fig Relative contribution of cell surface carbohydrate receptors on activation of PI3K/Akt and MEK/ERK signaling pathways. (A) Serum*-*starved MA104 cells were pretreated with NaIO_4_ (1 mM or 10 mM) for 30 min at 4°C and then incubated with the purified VP8* proteins of the RVA DS-1 or NCDV strains. The cell lysates were subjected to Western blot analysis for the detection of pAkt and pERK using the relevant antibodies. (B and C) Serum*-*starved MA104 cells were treated with antibodies against Hsc70 (B) and αVβ3 integrin (C) for 2 h at 37°C, and then incubated with purified VP5* protein (B) and purified VP7protein (C) of the RVA DS-1 and NCDV strains. The cell lysates were subjected to Western blot analysis for the detection of pAkt and pERK using the relevant antibodies. GAPDH was used as a loading control. The fold change of pAkt and pERK relative to GAPDH was determined by densitometric analysis and is indicated above each lane.
(TIF)

S12 Fig PI3K/Akt and MEK/ERK signaling pathways are not activated by crystal trypsin. (A and B) MA104 cells were mock-treated or treated with 10 μg/ ml crystal trypsin for the indicated time points. The cell lysates were then subjected to Western blot analysis to check the expression levels of pPI3K, PI3K, pAkt, Akt, pERK, and ERK. GAPDH was used as a loading control. The intensity of pPI3K, pAkt, and pERK relative to GAPDH were determined by densitometric analysis and is indicated above each lane.
(TIF)

S13 Fig Recombinant NSP1 and VP6 proteins of RVAs do not activate PI3K/Akt and MEK/ERK signaling pathways. Serum*-*starved MA104 cells were incubated with recombinant his-tagged NSP1 protein of the DS-1 strain (A) or the NCDV strain (B), or with his-tagged VP6 protein of the DS-1 strain (C) or the NCDV strain (D) at 10 μg/ml for the indicated time points. The cell lysates were subjected to Western blot analysis for the detection of pPI3K, pAkt, pERK, PI3K, Akt, and ERK using the relevant antibodies. GAPDH was used as a loading control. The fold change of pPI3K, pAkt, and pERK relative to GAPDH was determined by densitometric analysis and is indicated above each lane.
(TIF)
