# Supplementary material for: Activation of PI3K, Akt, and ERK during early rotavirus infection leads to V-ATPase-dependent endosomal acidification required for uncoating
Source: PLoS Pathog. 2018 Jan 19;14(1):e1006820. doi: 10.1371/journal.ppat.1006820 (PMC5792019; doi:10.1371/journal.ppat.1006820)
Supplement: S1 Table — (DOCX) [file ppat.1006820.s001.docx]

**S1 Table.** Oligonucleotide primers used in this study.

| Strain | Target gene | Sequence (5’ to 3’) | Region (nt) | Size (bp) |
| --- | --- | --- | --- | --- |
| DS-1 | VP8 | F: GT GGATCC *ATG* GCT TCA CTC ATT TAT AGA C | 10–31 | 690 |
|  |  | R: GC CTCGAG *TCA* TCT AGT ATT CTG AAT TGG TGG | 679–699 |  |
| NCDV | VP8 | F: GT GGATCC *ATG* GCT TCA CTC ATT TAT AGA C | 10–31 | 693 |
|  |  | R: GC CTCGAG *TCA* TCT CGT ATT TTG TAT TGG TGG | 682–702 |  |
| RRV | VP8 | F: GT GGATCC *ATG* GCT TCG CTC ATT TAT AGA C | 10–31 | 693 |
|  |  | R: GC CTCGAG *TTA* TCG TGT ATT CTG AAT CGG AGG T | 681–702 |  |
| DS-1 | VP5 | F: GT GGATCC *ATG* GCA CAA GTT AAT GAA | 748–762 | 1590 |
|  |  | R: GC CTCGAG *TCA* **ATG GTG ATG GTG ATG GTG** CAA CTT ACA TTG | 2323–2337 |  |
| NCDV | VP5 | F: GT GGATCC *ATG* GCA CAA CCT A AT CAA | 751–765 | 1590 |
|  |  | R: GCCTCGAG *TTA* **ATG GTG ATG GTG ATG GTG** CAA GCG ACA TTG | 2326–2340 |  |
| RRV | VP5 | F: GT GGATCC *ATG* GCT CAA GCG AAT GAA GAT | 748–768 | 1593 |
|  |  | R: GC CTCGAG *TTA* **ATG GTG ATG GTG ATG GTG** CAG TCT ACA CTG | 2318–2340 |  |
| DS-1 | VP7 | F: TA GGATCC *ATG* TAT GGT ATT GAA TAT ACC ACA ATT CTG ACC | 49–81 | 981 |
|  |  | R: ACCTCGAG *CTA* **ATG GTG ATG GTG ATG GTG** AAT TCT ATA ATA AAA AGC | 1009–1029 |  |
| NCDV | VP7 | F: TA GGATCC *ATG* TAT GGT ATT GAA TAT ACC ACA ATT CTA ATC TTC TTG ACA TCG | 49–93 | 981 |
|  |  | R: AC CTCGAG *CTA* **ATG GTG ATG GTG ATG GTG** CAC TCT ATA GTA GAA CGC | 1009–1029 |  |
| RRV | VP7 | F: GT GGATCC *ATG* GAA TGG TAT TGA ATA TAC CAC AG | 31–56 | 982 |
|  |  | R: GC CTCGAG *CTA* **ATG GTG ATG GTG ATG GTG** TAT TCT ATA ATA | 989–1012 |  |
| DS-1 | NSP1 | F: CACA ccatgg CG AAA AGT CTT GTG | 11–33 | 1482 |
|  |  | R: AAT CTCGAG *TCA* TTC AAT ATC GGA T | 1471–1492 |  |
| NCDV | NSP1 | F: CACA ccatgg CG ACT TTT AAA GAC | 32–55 | 1476 |
|  |  | R: AAT CTCGAG *TTA* CTC AAC ATC TGA A | 1481–1507 |  |
| DS-1 | VP6^a^ | F: CACA ccatgg AT GTT TTA TAT TCA TTA TCA A | 1–25 | 1194 |
|  |  | R: AAT CTCGAG *TCA* TTT AAC AAG CAT GCT TCT AAT | 1171–1194 |  |
| NCDV | VP6^a^ | F: CACA ccatgg AT GTC CTG TAT TCC T TGT CAA | 24–42 | 1194 |
|  |  | R: AATCTCGAG *TCA* TTT GAC TAG CAT GCT TCT AAT G | 1193–1217 |  |
| DS-1 | VP6^b^ | F: TAG ACC GAA TAA TGT CGA AGT AGA  R: GAC TCA CAA ACT GCA GAT TCA A | 785–808  1000–1021 | 237 |
| NCDV | VP6^b^ | F: TAG ACC AAA TAA CGT TGA AGT TGA  R: GAT TCA CAA ACT GCA GAT TCA A | 785–808  1000–1021 | 237 |

The underlined capital letters represent restrictions enzyme sites (forward primer: BamHI; reverse primer: XhoI). The underlined small letters represent NcoI restriction enzyme site. The start or stop codons are in italics. The bold represents the polyhistidine-tag (6xHis tag) sequences.

^a^For recombinant protein expression.

^b^For qRT-PCR.
