# Supplementary material for: Activation of PI3K, Akt, and ERK during early rotavirus infection leads to V-ATPase-dependent endosomal acidification required for uncoating
Source: PLoS Pathog. 2018 Jan 19;14(1):e1006820. doi: 10.1371/journal.ppat.1006820 (PMC5792019; doi:10.1371/journal.ppat.1006820)
Supplement: S2 Table — (DOCX) [file ppat.1006820.s002.docx]

**S2 Table.** Sequences of siRNAs against target molecules and scrambled siRNA used in this study.

| siRNA | | Sequence (5’ to 3’) |
| --- | --- | --- |
| Rab5 | A | Sense: GCAAGUCCUAACAUUGUAAtt  Antisense: UUACAAUGUUAGGACUUGCtt |
|  | B | Sense: CCAAAGAAUGAACCACAAAtt  Antisense: UUUGUGGUUCAUUCUUUGGtt |
|  | C | Sense: GUACCCGUAAUUUGUAACAtt  Antisense: UGUUACAAAUUACGGGUACtt |
| Rab7 | A | Sense: GGAAGACAUCACUCAUGAAtt  Antisense: UUCAUGAGUGAUGUCUUCCtt |
|  | B | Sense: CCAGUAUGUGAAUAAGAAAtt  Antisense: UUUCUUAUUCACAUACUGGtt |
|  | C | Sense: GCGUUCUGGUAUUUGAUGUtt  Antisense: ACAUCAAAUACCAGAACGCtt |
| PI 3-kinase p85α | A | Sense: CAGCUCUGAUAAUACUGAAtt  Antisense: UUCAGUAUUAUCAGAGCUGtt |
|  | B | Sense: GCAUGGUGAUUAUACUCUUtt  Antisense: AAGAGUAUAAUCACCAUGCtt |
|  | C | Sense: GGAUCAAGUUGUCAAAGAAtt  Antisense: UUCUUUGACAACUUGAUCCtt |
| MEK-1 | A | Sense: AAGCAGAAGGUGGGAGAACtt  Antisense: GUUCUCCCACCUUCUGCUUtt |
| MEK-2 | A | Sense: AGAGGCCAAGAGGAUUCCCtt  Antisense: GGGAAUCCUCUUGGCCUCUtt |
|  | B | Sense: GAUGCUCACAAACCACACCtt  Antisense: GGUGUGGUUUGUGAGCAUCtt |
| Scramble | A | r(UUCUCCGAACGUGUCACGU)d(TT) |
