# Supplementary figures and images for: Activation of PI3K, Akt, and ERK during early rotavirus infection leads to V-ATPase-dependent endosomal acidification required for uncoating
Source: PLoS Pathog. 2018 Jan 19;14(1):e1006820. doi: 10.1371/journal.ppat.1006820 (PMC5792019; doi:10.1371/journal.ppat.1006820)

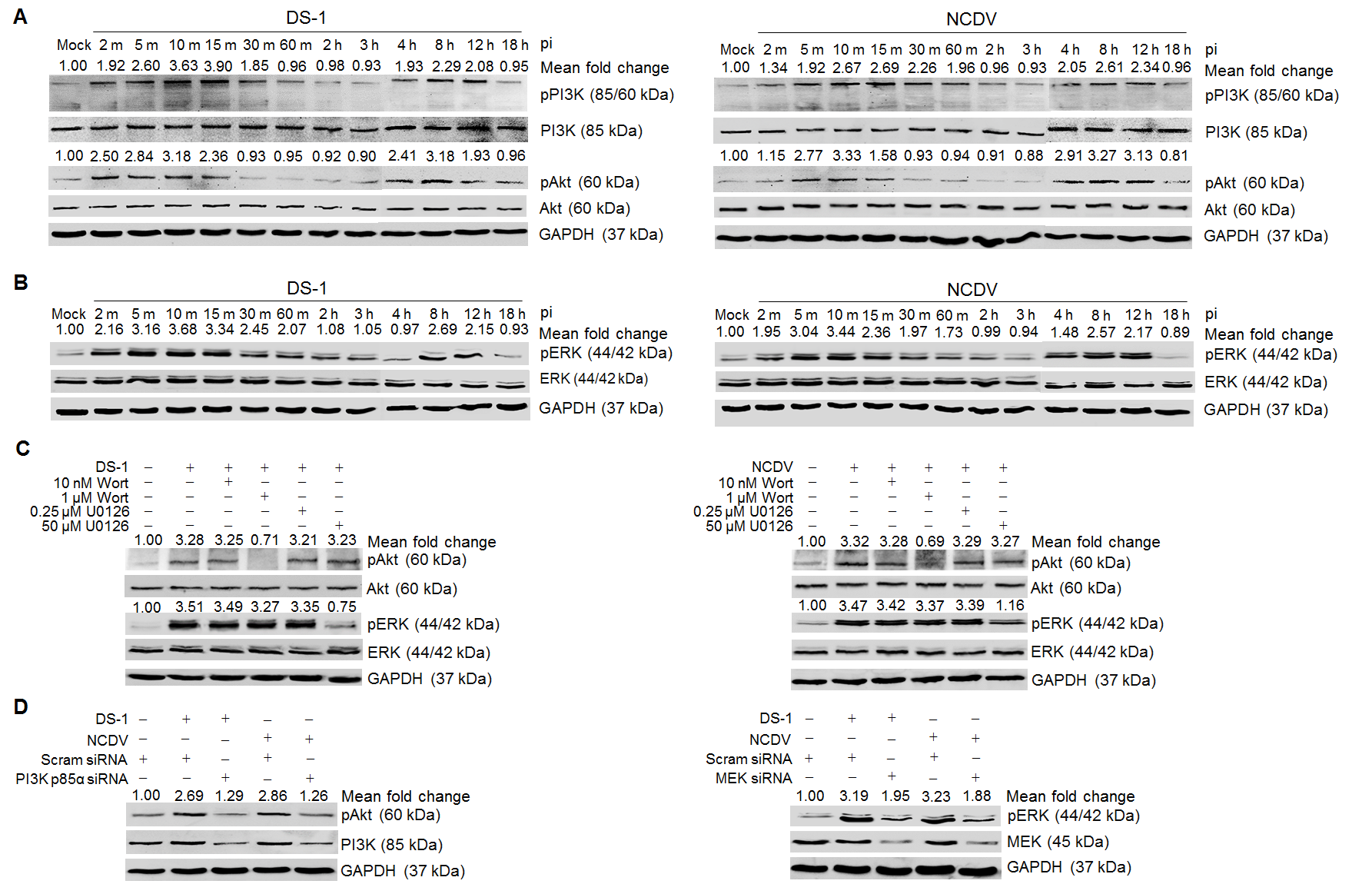

Supplement: S1 Fig — (A and B) Caco-2 cells were mock-infected or infected with the DS-1 or NCDV strains (MOI = 10 FFU/cell) for the indicated time. The cells were then harvested at the indicated time points. The cell lysates were subjected to Western blot analysis to check the expression levels of phosphorylated PI3K (pPI3K), PI3K, pAkt, Akt, pERK, and ERK using the relevant antibody. GAPDH was used as a loading control. (C) Caco-2 cells were mock treated or pretreated with wortmannin or U0126 at the indicated doses for 1 h at 37°C, followed by infection with DS-1 and NCDV. Cell lysates were harvested at 5 mpi and the expression levels of pAkt, Akt, pERK, and ERK were evaluated by Western blot analysis using the relevant antibody. GAPDH was used as a loading control. (D) Caco-2 cells were transfected with scrambled siRNA or siRNAs specific for PI3K p85α or MEK, and then infected with either the human RVA DS-1 or the bovine RVA NCDV strains (MOI = 10 FFU/cell). The cell lysates were subjected to Western blot analysis to check the expression levels of pAkt, Akt, pERK, and ERK using the corresponding antibody. GAPDH was used as a loading control. The intensity of pPI3K, pAkt, and pERK relative to GAPDH was determined by densitometric analysis and is indicated above each lane. (TIF) [file ppat.1006820.s003.tif]

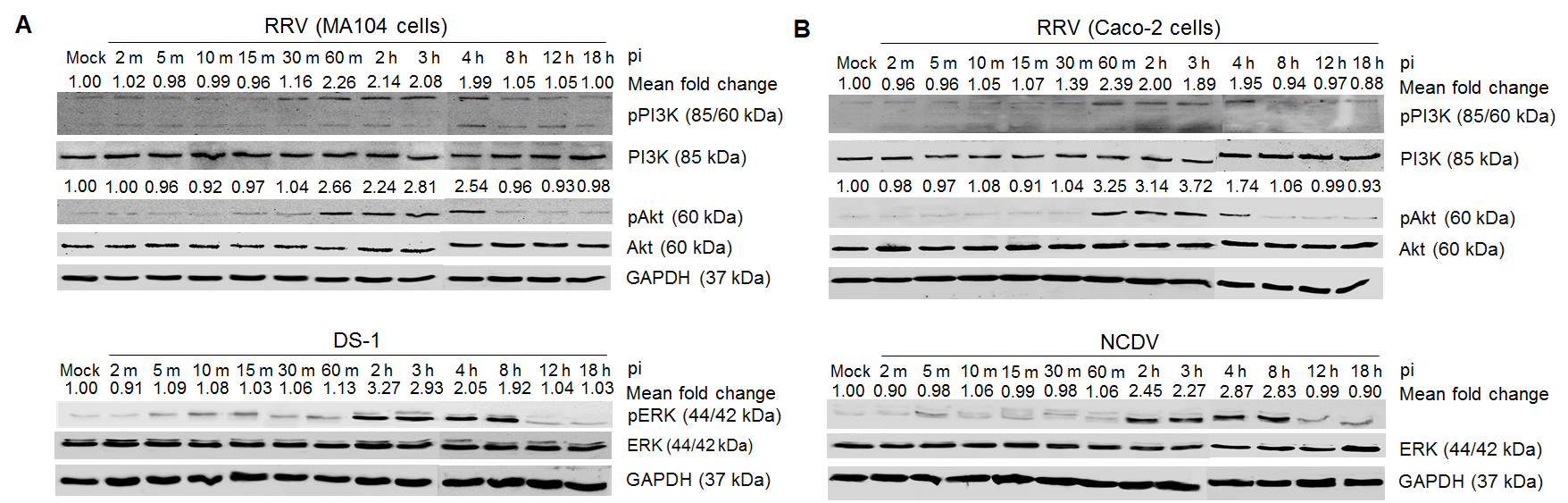

Supplement: S2 Fig — MA104 cells (A) and Caco-2 cells (B) were infected with the simian RVA strain RRV (MOI = 10 FFU/cell) for the indicated time points. The cell lysates were subjected to Western blot analysis to check the expression levels of phosphorylated PI3K (pPI3K), PI3K, pAkt, Akt, pERK, and ERK using the relevant antibody. GAPDH was used as a loading control. The intensity of pPI3K, pAkt, and pERK relative to GAPDH was determined by densitometric analysis and is indicated above each lane. (TIF) [file ppat.1006820.s004.tif]

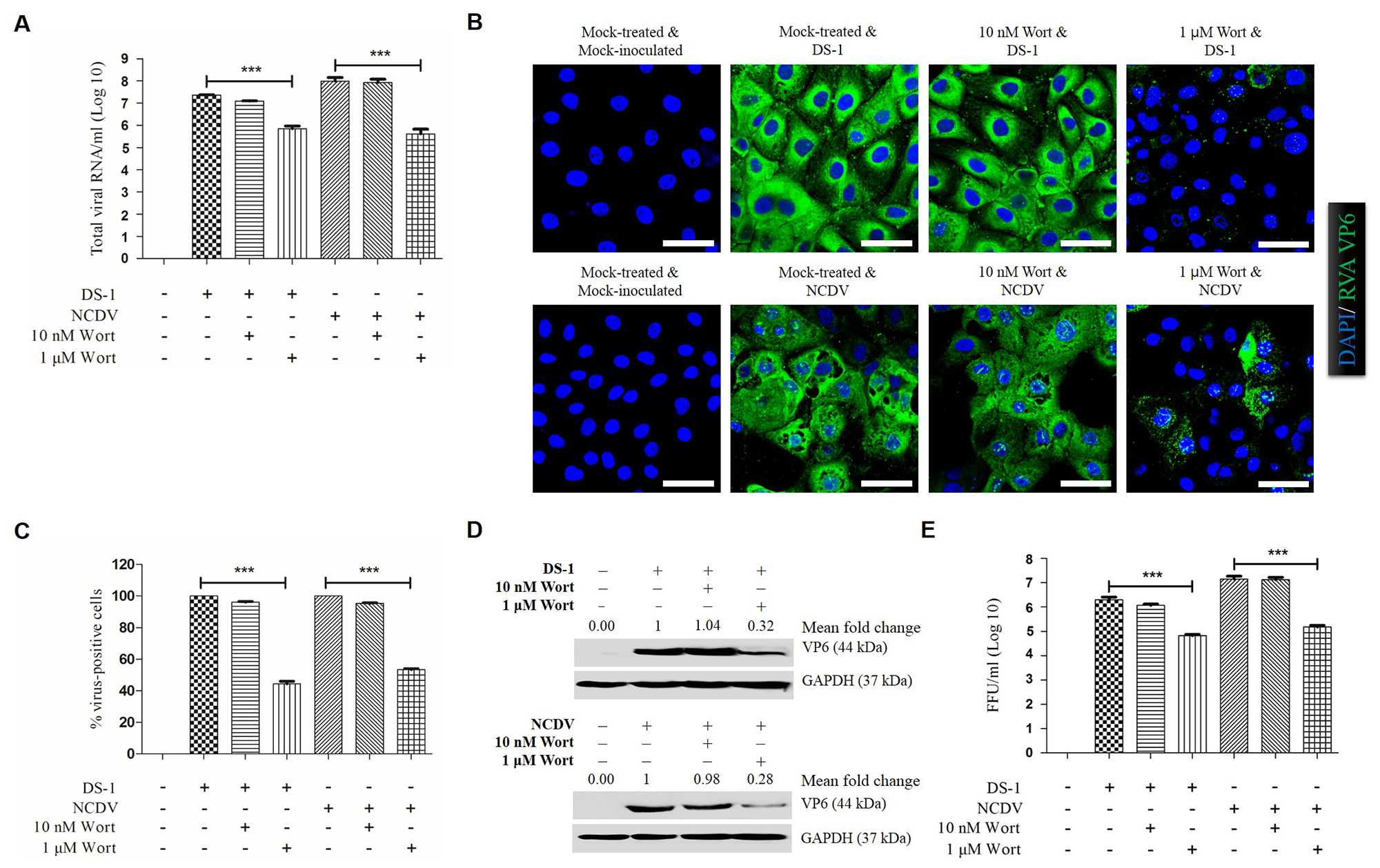

Supplement: S3 Fig — MA104 cells were pretreated with a non-cytotoxic concentration of wortmannin for 1 h at 37°C and then infected with the RVA strains DS-1 and NCDV (MOI = 10 FFU/cell) for 8 h. The total viral RNA (A), antigen-positive cells (using anti-RVA VP6 Mab) (B and C), and VP6 protein (D) were determined by real-time RT-PCR, immunofluorescence, and Western blot analyses, respectively. GAPDH was used as a loading control. The intensity of pPI3K, pAkt, and pERK relative to GAPDH was determined by densitometric analysis and is indicated above each lane. (E) The virus titer was determined by cell culture immunofluorescence assay using cell lysates produced by 3 cycles of freezing and thawing; the results are expressed as fluorescent focus forming unit (FFU). All experiments were performed in triplicate; panel B shows a representative set of results. Data are presented as means ± standard error of the mean from three independent experiments. Differences were evaluated using the One-Way ANOVA. *p<0.05; **p<0.001; ***p<0.0001. The scale bars in panel B correspond to 20 μm. (TIF) [file ppat.1006820.s005.tif]

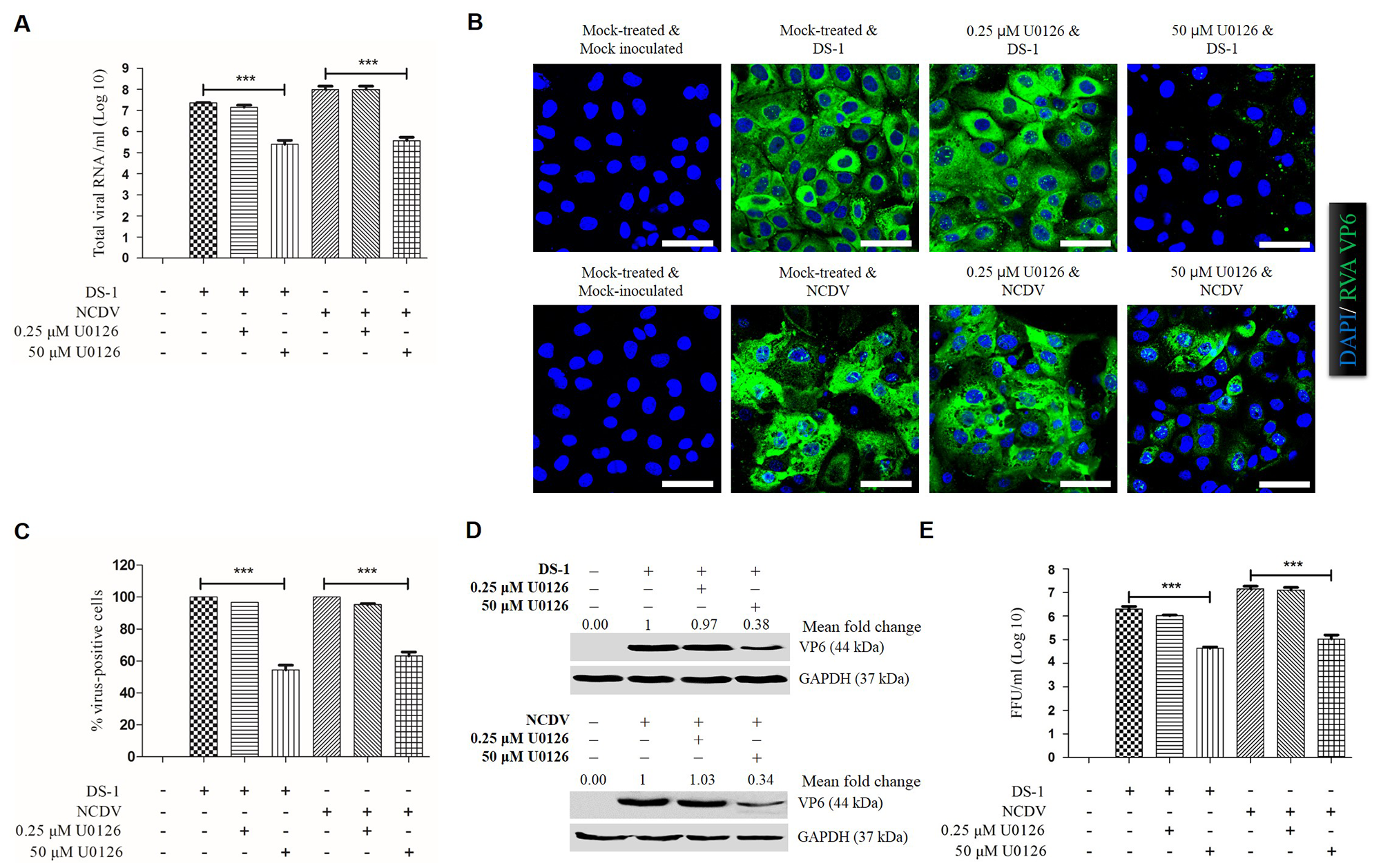

Supplement: S4 Fig — MA104 cells were pretreated with a non-cytotoxic concentration of U0126 for 1 h at 37°C and then infected with the RVA strains DS-1 and NCDV (MOI = 10 FFU/cell) for 8 h. The total viral RNA (A), antigen-positive cells (using anti-RVA VP6 Mab) (B and C), and VP6 protein (D) were determined by real-time RT-PCR, immunofluorescence, and Western blot analyses, respectively. GAPDH was used as a loading control. The intensity of pPI3K, pAkt, and pERK relative to GAPDH was determined by densitometric analysis and is indicated above each lane. (E) The virus titer was determined by cell culture immunofluorescence assay using cell lysates produced by 3 cycles of freezing and thawing and are expressed as FFU. All experiments were performed in triplicate and panel B shows a representative set of results. Data are presented as means ± standard error of the mean from three independent experiments. Differences were evaluated by the One-Way ANOVA. *p<0.05; **p<0.001; ***p<0.0001. The scale bars in panel B correspond to 20 μm. (TIF) [file ppat.1006820.s006.tif]

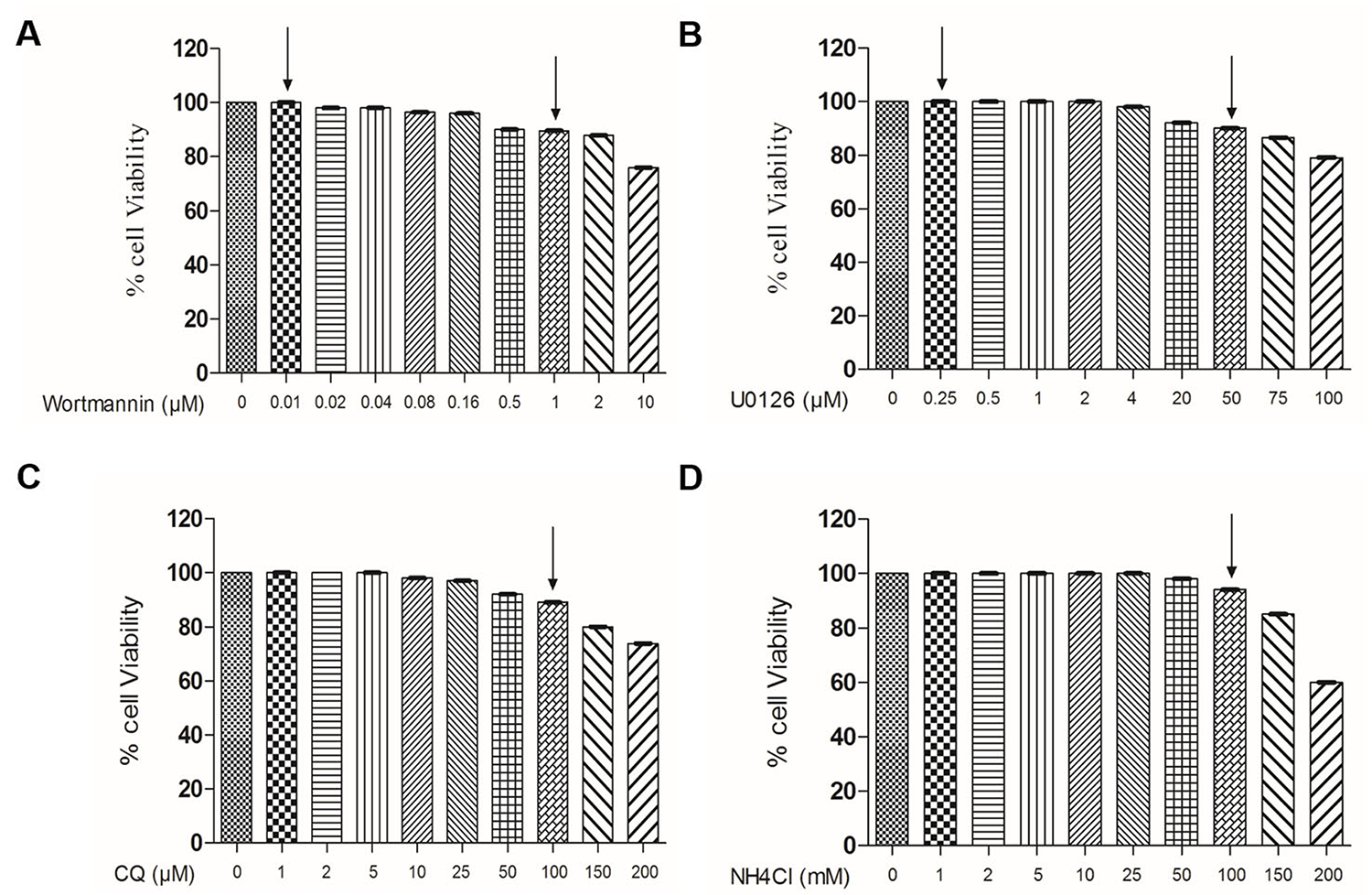

Supplement: S5 Fig — (A-D) MA104 cells grown in 96-well plates were incubated with various concentrations of the indicated chemicals in triplicate for 24 h at 37°C. Afterwards, the chemicals-containing media was thoroughly removed and replaced with 200 μl of MTT solution for 4 h at 37°C. Each well was incubated with 100 μl of DMSO for 10 min at room temperature. Cell viability was measured using an ELISA reader at an OD value of 570 nm. The arrows indicate the concentrations used in this study. (TIF) [file ppat.1006820.s007.tif]

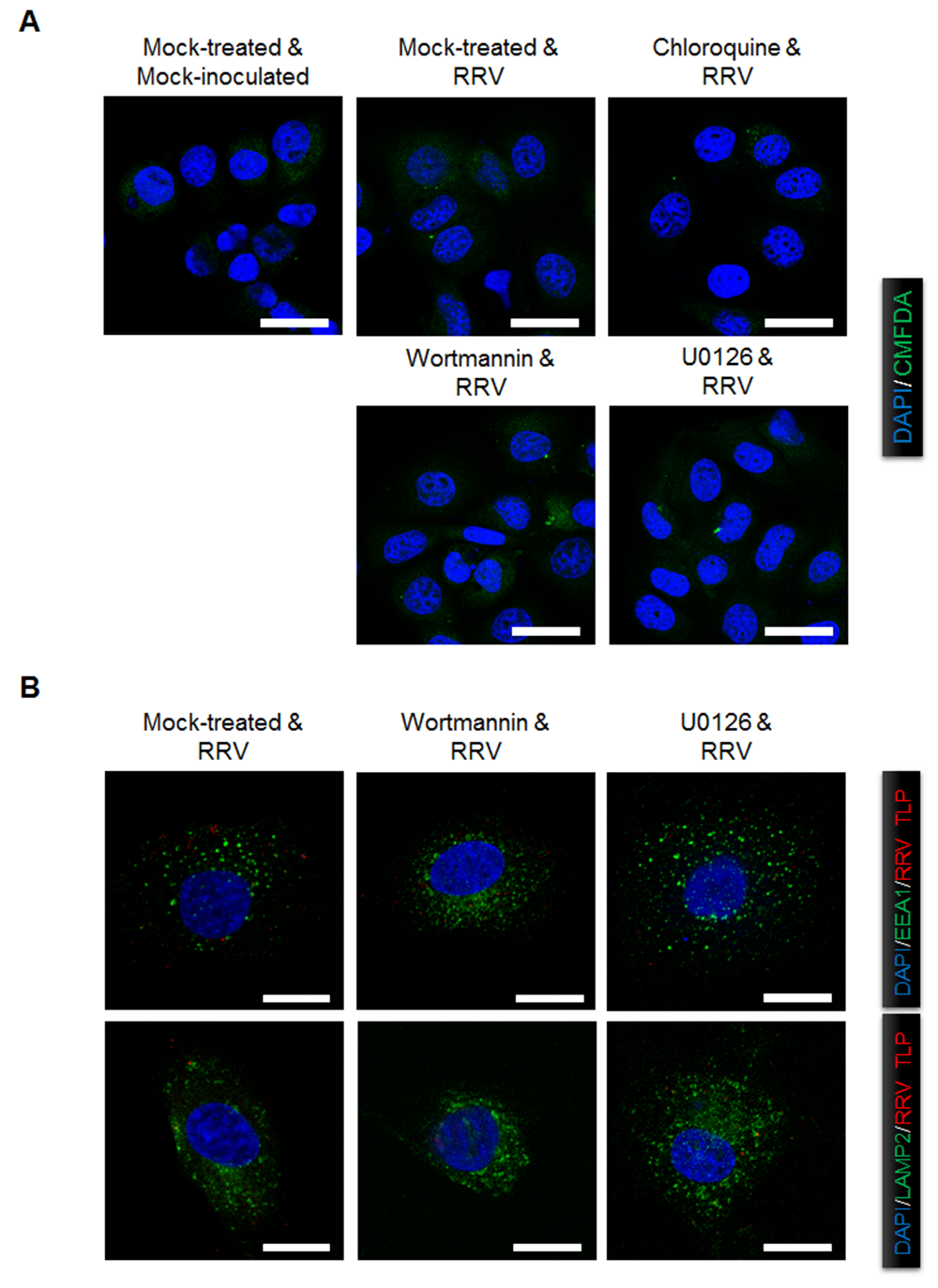

Supplement: S6 Fig — (A) MA104 cells were pretreated with or without chloroquine, wortmannin, or U0126 for 1 h at 37°C and subsequently infected with the RRV strain (MOI = 10 FFU/cell) for 30 min at 37°C. Cells were then incubated for 30 min with CMFDA (10 μM) to visualize acidification of intracellular compartments followed by 30 min in serum-free media. (B) MA104 cells were pretreated with or without wortmannin or U0126 for 1 h at 37°C and then infected with the RRV strain (MOI = 10 FFU/cell) for 3 h. After fixation and permeabilization, the cells were prepared for confocal microscopy using anti-TLP, anti-EEA1, and anti-LAMP2 antibodies, and the relevant secondary antibodies. Representative images are shown. The scale bars correspond to 10 μm (A) and 5 μm (B). (TIF) [file ppat.1006820.s008.tif]

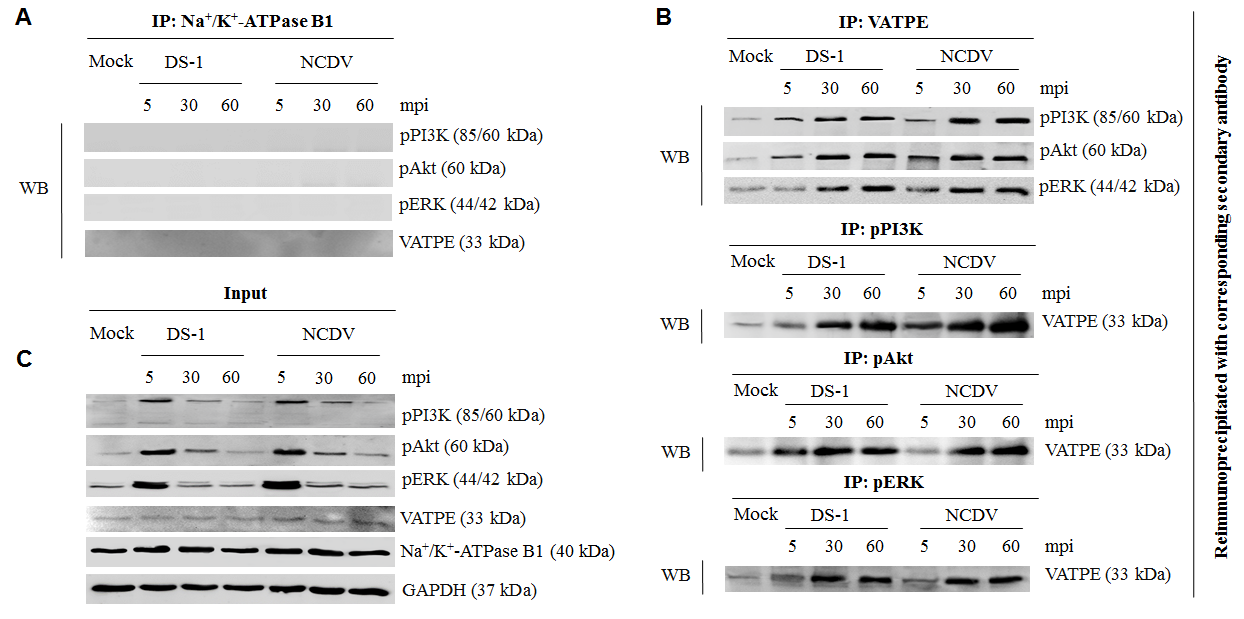

Supplement: S7 Fig — (A) Serum-starved MA104 cells were inoculated with RVA DS-1 or NCDV (MOI = 10 FFU/cell) for the indicating time. Subsequently, the cell lysates were immunoprecipitated using antibody specific for the Na+/K+-ATPase B1. (B) To rule out entrapment of any of the V-ATPase, pPI3K, pAkt, and pERK during immunoprecipitation, the pre-cleared cell lysates were incubated with antibodies against the V-ATPase, pPI3K, pAkt, and pERK. Each reaction mixture was then incubated with secondary antibodies specific for each primary antibody. The co-immunoprecipitated products and control inputs (C) were analyzed by Western blot analysis to detect pPI3K, pAkt, pERK, V1 subunit E, and Na+/K+-ATPase B1 using the relevant antibodies. GAPDH was used as a loading control. (TIF) [file ppat.1006820.s009.tif]

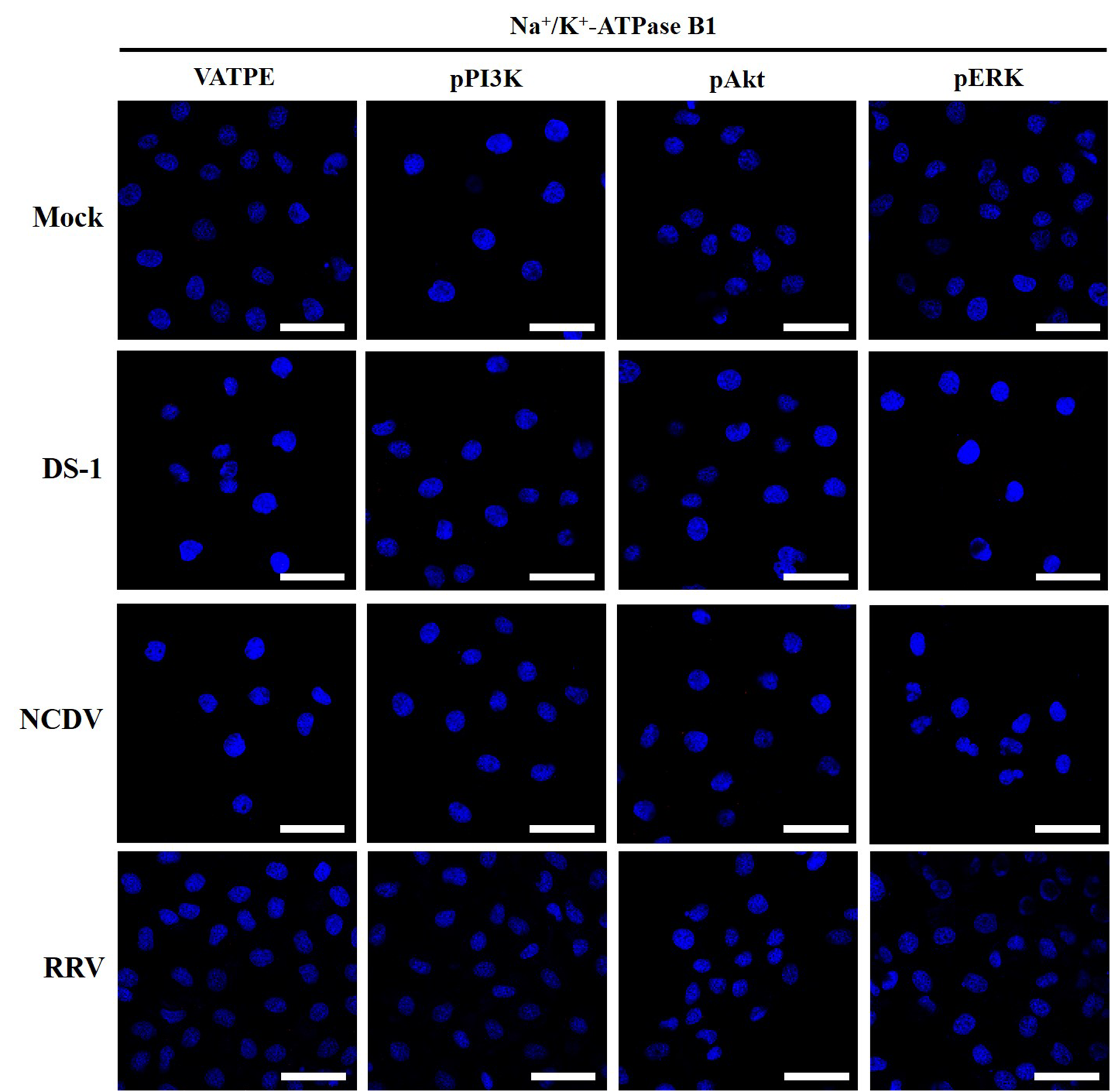

Supplement: S8 Fig — Serum-starved MA104 cells were either mock-inoculated or inoculated with the RVA strains DS-1, NCDV, and RRV (MOI = 10 FFU/cell). Subsequently, the cells were fixed, permeabilized and incubated with two primary antibodies, the primary antibody against Na+/K+-ATPase B1 protein and primary V-ATPase E subunit, pPI3K, pAkt, or pERK antibodies overnight at 4°C. The Duolink PLA was performed as described in the Materials and Methods section and the signals are represented as red dots. Representative images are shown. The scale bars correspond to 20 μm. (TIF) [file ppat.1006820.s010.tif]

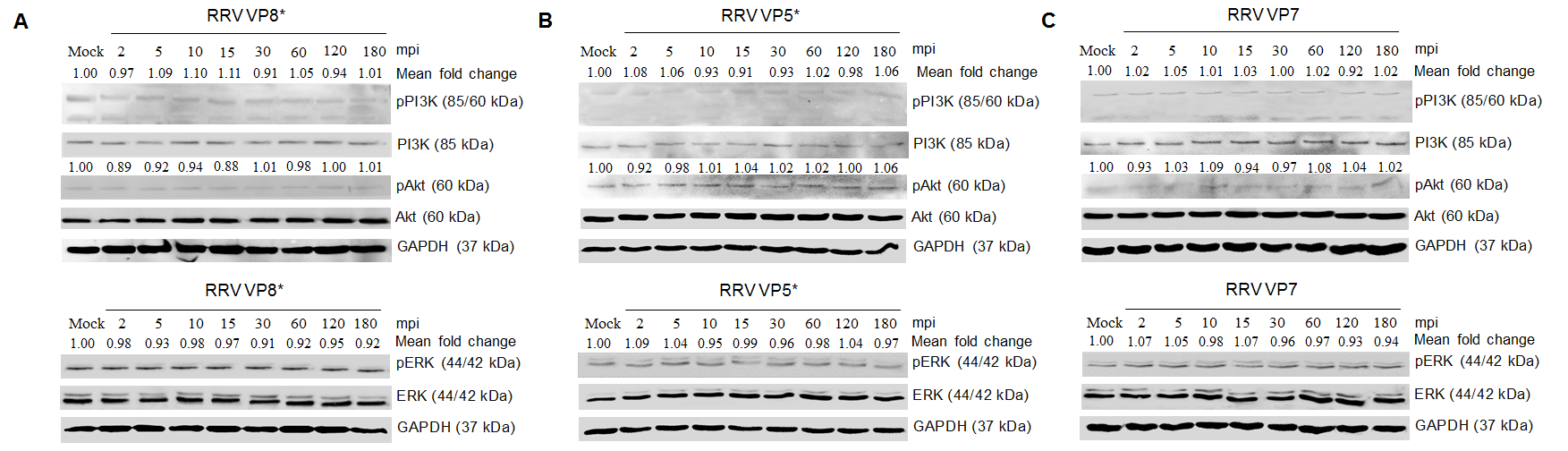

Supplement: S9 Fig — Serum-starved MA104 cells were incubated with the recombinant GST-fused VP8* (A) or his-tagged VP5* (B) or VP7 proteins (C) of the RRV strain at 10 μg/ml for the indicated time points. The cell lysates were subjected to Western blot analysis for the detection of pPI3K, pAkt, pERK, PI3K, Akt, and ERK using the relevant antibodies. GAPDH was used as a loading control. The fold change of pPI3K, pAkt, and pERK relative to GAPDH was determined by densitometric analysis and is indicated above each lane. (TIF) [file ppat.1006820.s011.tif]

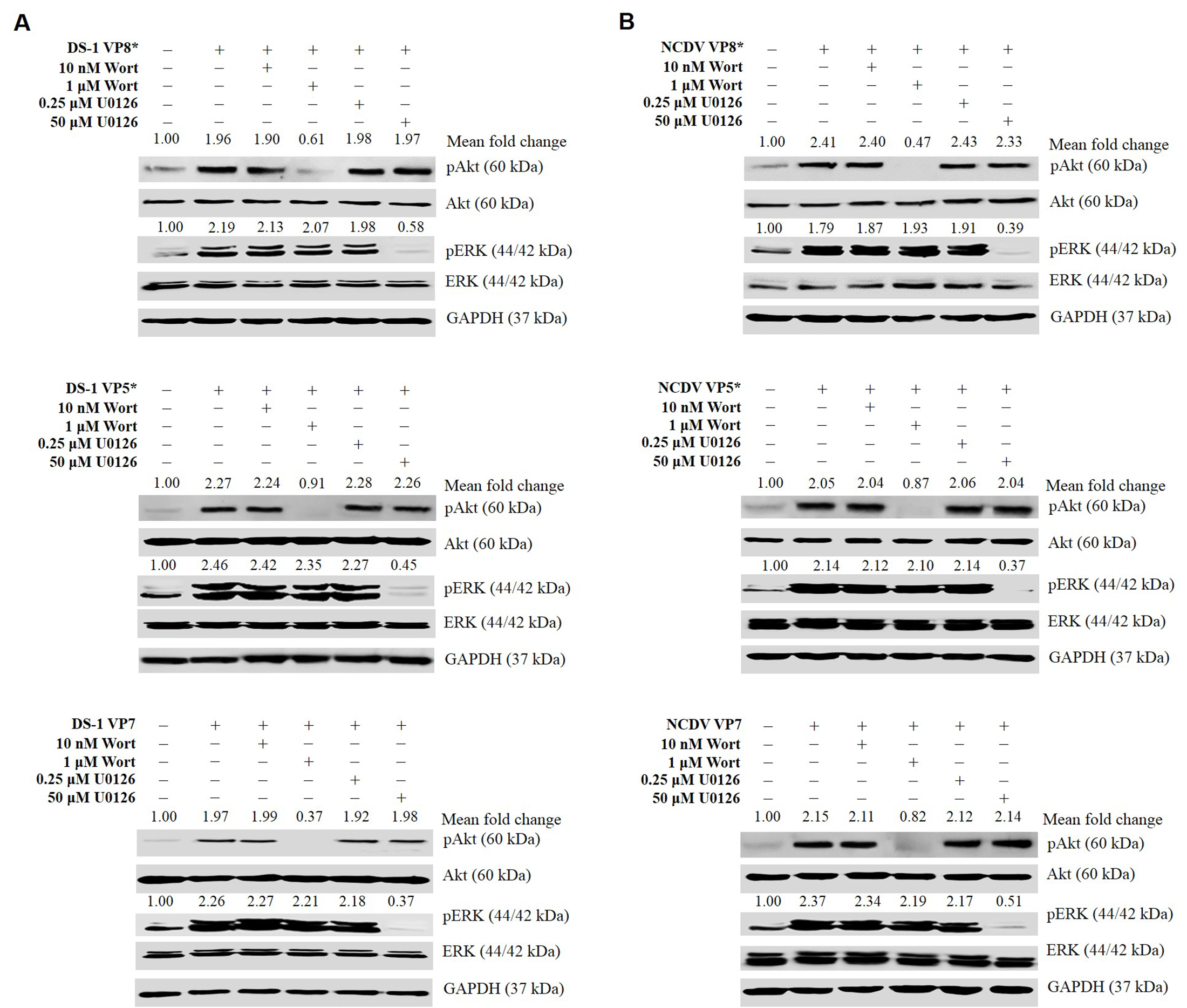

Supplement: S10 Fig — MA104 cells were mock-treated or treated with wortmannin or U0126 and then incubated with purified VP8*, VP5*, or VP7 proteins of RVA DS-1 (A) and NCDV (B) strains at 10 μg/ml. Cell lysates were harvested at 5 mpi and the expression levels of pAkt, Akt, pERK, and ERK were evaluated by Western blot analysis. GAPDH was used as a loading control. The intensity of pPI3K, pAkt, and pERK relative to GAPDH were determined by densitometric analysis and is indicated above each lane. (TIF) [file ppat.1006820.s012.tif]

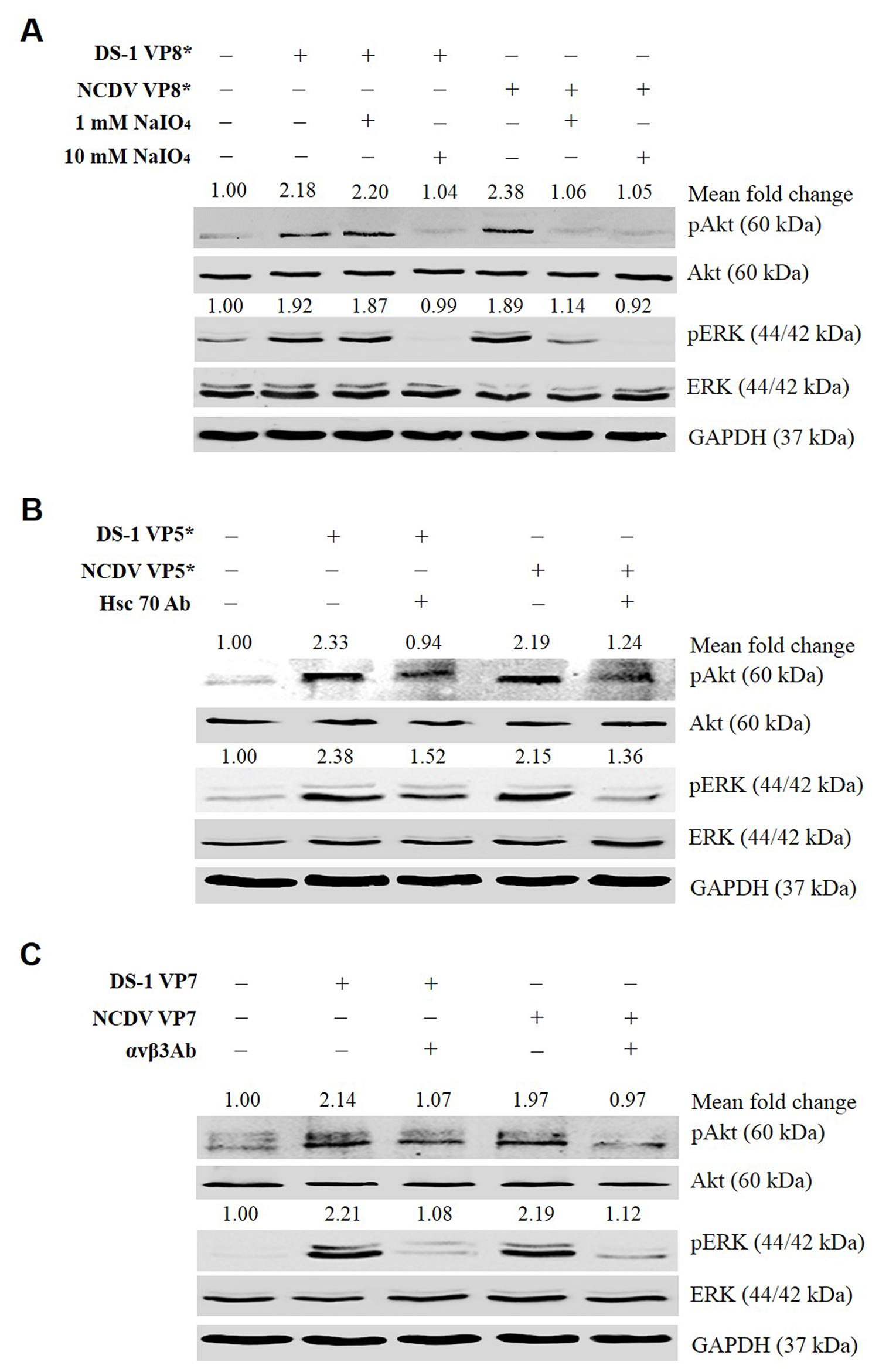

Supplement: S11 Fig — (A) Serum-starved MA104 cells were pretreated with NaIO4 (1 mM or 10 mM) for 30 min at 4°C and then incubated with the purified VP8* proteins of the RVA DS-1 or NCDV strains. The cell lysates were subjected to Western blot analysis for the detection of pAkt and pERK using the relevant antibodies. (B and C) Serum-starved MA104 cells were treated with antibodies against Hsc70 (B) and αVβ3 integrin (C) for 2 h at 37°C, and then incubated with purified VP5* protein (B) and purified VP7protein (C) of the RVA DS-1 and NCDV strains. The cell lysates were subjected to Western blot analysis for the detection of pAkt and pERK using the relevant antibodies. GAPDH was used as a loading control. The fold change of pAkt and pERK relative to GAPDH was determined by densitometric analysis and is indicated above each lane. (TIF) [file ppat.1006820.s013.tif]

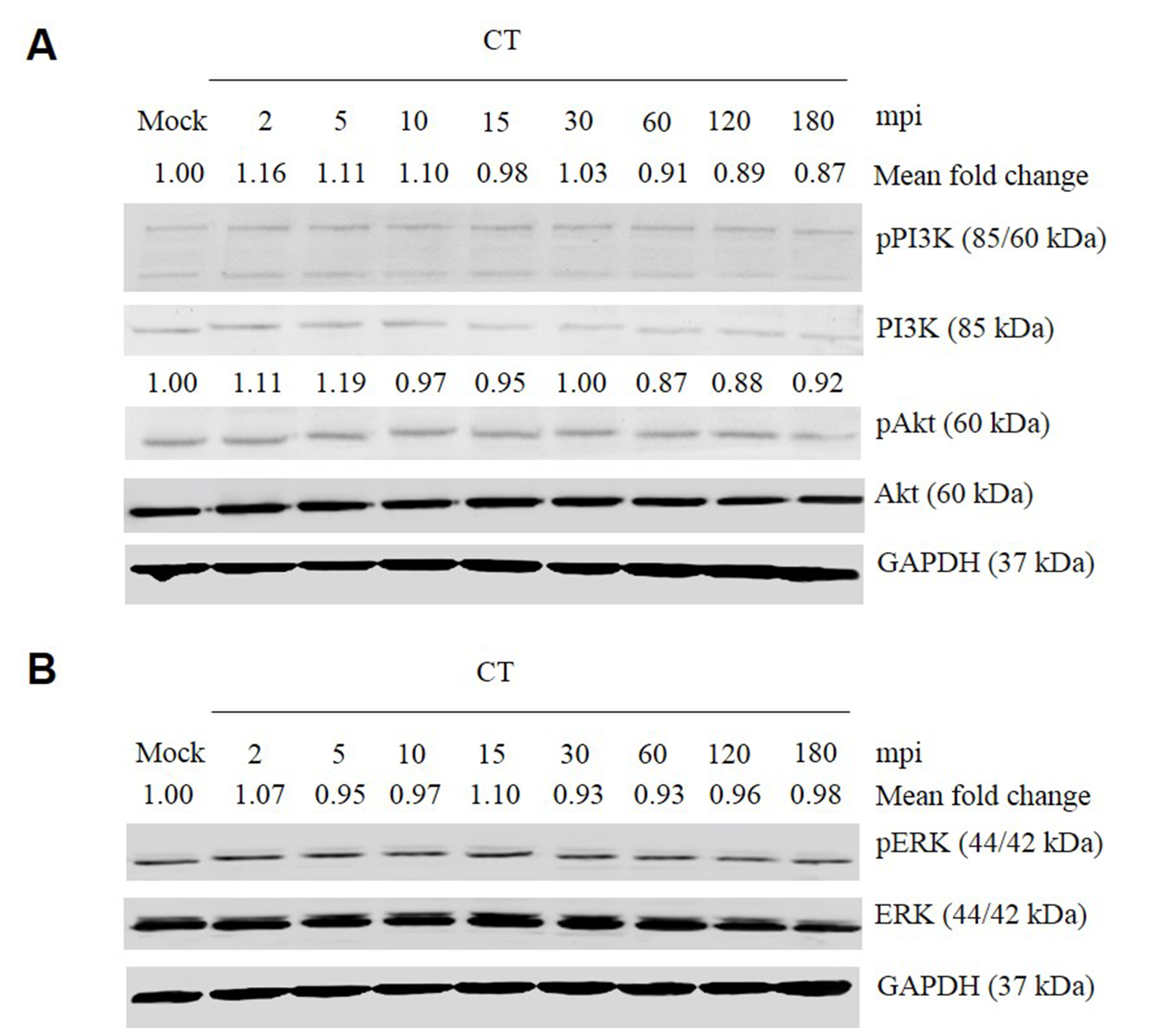

Supplement: S12 Fig — (A and B) MA104 cells were mock-treated or treated with 10 μg/ ml crystal trypsin for the indicated time points. The cell lysates were then subjected to Western blot analysis to check the expression levels of pPI3K, PI3K, pAkt, Akt, pERK, and ERK. GAPDH was used as a loading control. The intensity of pPI3K, pAkt, and pERK relative to GAPDH were determined by densitometric analysis and is indicated above each lane. (TIF) [file ppat.1006820.s014.tif]

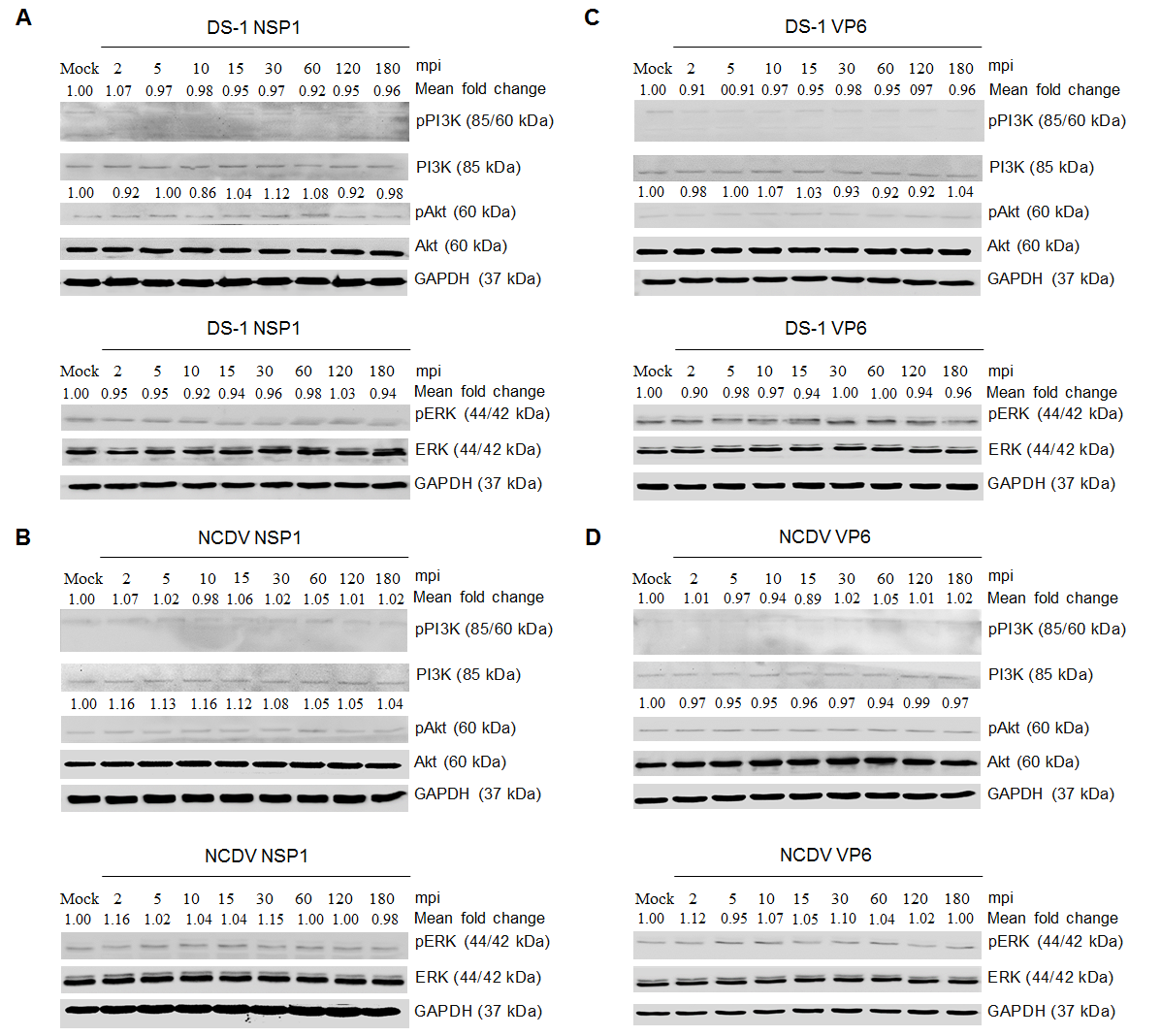

Supplement: S13 Fig — Serum-starved MA104 cells were incubated with recombinant his-tagged NSP1 protein of the DS-1 strain (A) or the NCDV strain (B), or with his-tagged VP6 protein of the DS-1 strain (C) or the NCDV strain (D) at 10 μg/ml for the indicated time points. The cell lysates were subjected to Western blot analysis for the detection of pPI3K, pAkt, pERK, PI3K, Akt, and ERK using the relevant antibodies. GAPDH was used as a loading control. The fold change of pPI3K, pAkt, and pERK relative to GAPDH was determined by densitometric analysis and is indicated above each lane. (TIF) [file ppat.1006820.s015.tif]
